# Pyruvate kinase L/R links metabolism dysfunction to neuroendocrine differentiation of prostate cancer by ZBTB10 deficiency

**DOI:** 10.1038/s41419-022-04694-z

**Published:** 2022-03-19

**Authors:** Yu-Ching Wen, Wei-Yu Chen, Van Thi Ngoc Tram, Hsiu-Lien Yeh, Wei-Hao Chen, Kuo-Ching Jiang, Wassim Abou-Kheir, Jiaoti Huang, Michael Hsiao, Yen-Nien Liu

**Affiliations:** 1 Department of Urology, Wan Fang Hospital, Taipei Medical University, Taipei, 116 Taiwan; 2 Department of Urology, School of Medicine, College of Medicine, Taipei Medical University, Taipei, 110 Taiwan; 3 TMU Research Center of Urology and Kidney, Taipei Medical University, Taipei, 110 Taiwan; 4 Department of Pathology, Wan Fang Hospital, Taipei Medical University, Taipei, 116 Taiwan; 5 Department of Pathology, School of Medicine, College of Medicine, Taipei Medical University, Taipei, 110 Taiwan; 6 International Ph.D Program in Medicine, College of Medicine, Taipei Medical University, Taipei, 110 Taiwan; 7 General Education Development Center, Hsin Sheng Junior College of Medical Care and Management, Taoyuan, 325 Taiwan; 8 Department of Anatomy, Cell Biology and Physiological Sciences Faculty of Medicine, American University of Beirut, Beirut, 1107-2020 Lebanon; 9 Department of Pathology, Duke University Medical Center, Durham, NC 27710 USA; 10 Genomics Research Center, Academia Sinica, Taipei, 115 Taiwan

**Keywords:** Prostate cancer, Oncogenes, Prognostic markers

## Abstract

Neuroendocrine differentiation (NED) frequently occurs in androgen-deprivation therapy (ADT)-resistant prostate cancer (PCa) and is typically associated with metabolic pathway alterations, acquisition of lineage plasticity, and malignancy. There is no conventional therapeutic approach for PCa patients with NED pathologic features because the molecular targets are unknown. Here, we evaluated the regulatory mechanism of NED-associated metabolic reprogramming induced by ADT. We detected that the loss of the androgen-responsive transcription factor, zinc finger, and BTB domain containing 10 (ZBTB10), can activate pyruvate kinase L/R (PKLR) to enhance a NED response that is associated with glucose uptake by PCa cells. PKLR exhibits a tumor-promoting effect in PCa after ADT, but ZBTB10 can compensate for the glucose metabolism and NED capacity of PKLR through the direct transcriptional downregulation of *PKLR*. Targeting PKLR by drug repurposing with FDA-approved compounds can reduce the aggressiveness and NED of ADT-resistant PCa. We demonstrated that PKLR acts as a modulator to activate NED in PCa enhancement by loss of ZBTB10, thereby enabling PCa cells to mount a glycolysis response essential for therapeutic resistance. Our findings highlight the broad relation between NED and metabolic dysfunction to provide gene expression-based biomarkers for NEPC treatment.

## Introduction

Prostate cancer (PCa) represents a major cause of urologic-related morbidity and mortality in men worldwide [1]. The androgen receptor (AR) plays an important role in the basic mechanisms underlying PCa initiation and progression [2]. Therapeutic interventions with androgen-deprivation therapy (ADT) may lead to the development of metastatic castration-resistant PCa (CRPC) by activating androgen-independent pathways [3]. A subset of tumor cells from CRPC patients receiving ADT or current AR pathway inhibition (ARPI) therapy may progress to an AR-negative phenotype, and this is considered to result from an accumulation of neuroendocrine (NE) and stem cell characteristics [4]. The most important histological variation of NE-like PCa is NE differentiation (NED) PCa (NEPC) or small-cell NE PCa (SCPC), which shows high metastatic tendency and lineage plasticity [5]. NED occurs in 10–20% of CRPC cases, but not in all ADT patients [6]; however, with the widespread adoption of powerful AR-targeting drugs for PCa, NEPC may become more common with a clinically significant increase. Although chemotherapeutic strategies often involve management to increase the efficacy of NEPC treatment [7], currently available chemotherapeutic drugs still have side effects and do not offer a complete cure.

Activation of glucose metabolic pathways is very common during malignant transformation in PCa [8]. Cancer cells usually upregulate genes that encode glycolytic enzymes and pyruvate kinase, which leads to significantly higher glucose uptake and metabolic transformation to compensate for the loss of adenosine triphosphate (ATP) [9]. Pyruvate kinase L/R (PKLR) is a vital kinase that catalyzes the irreversible trans-phosphorylation between phosphoenolpyruvate (PEP) and adenosine diphosphate to generate pyruvate and ATP [10]. PKLR is required to maintain levels of major endogenous antioxidants and glutathione and is important in colorectal cancer cell survival [11]. In gallbladder cancer, PKLR promotes the Warburg effect to enhance cancer progression [12]. PKLR was also shown to be a predictive marker of peritoneal metastatic gastric cancer [13]. Inhibition of PKLR leads to decreased glucose uptake and decreased mitochondrial activity in hepatocellular carcinoma [14]. However, the mechanism of PKLR-regulated therapeutic resistance and NED progression in PCa remains unclear. Dysregulation of the AR signaling pathway in PCa cells revealed induction of their metabolic rewiring [15]. We sought to understand the metabolic basis of PKLR that influences the NED properties of tumors to provide a foundation for developing effective targeted strategies for PCa.

As the incidence of NEPC has increased following potent ARPI treatment [16], regulatory pathways involved in ARPI-induced activation of glycolytic enzymes urgently need to be investigated. PKLR is one of the key regulators of metabolic reprogramming supporting energy production [10], and it is important that we further understand the molecular basis of PKLR and define its role in the development of NED and therapeutic resistance of PCa. In this study, we investigated the role of PKLR in PCa and established the molecular basis of the tumor-promoting effects of PKLR that are associated with NEPC development after AR-targeted therapy. We provide evidence showing that loss of the androgen-responsive transcription factor, zinc finger, and BTB domain containing 10 (ZBTB10), is associated with PKLR upregulation and the NED of PCa. We demonstrated that overexpression of ZBTB10 can suppress the glycolysis and oncogenic effects of PKLR, indicating the tumor-suppressive role of ZBTB10 in PCa. We also showed that pharmacological inhibition of PKLR decreases tumor growth and NED progression in multiple in vitro and in vivo models of ADT-resistant and NE-like PCa cells, pointing to a potential approach for treating therapeutic-resistant PCa.

## Results

### Upregulation of PKLR is involved in NED of PCa after ADT

**Fig. 1:**
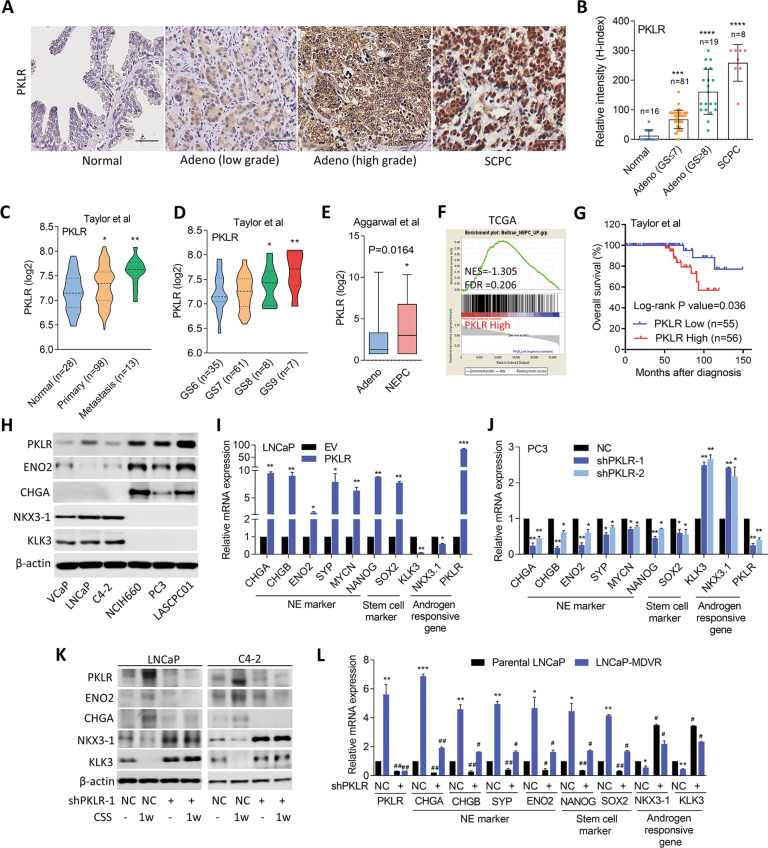
Upregulation of PKLR in PCa is associated with NED progression. **A**, **B** IHC staining (**A**) and representative intensity (**B**; by H-score analysis) of PKLR in PCa TMA sections containing normal tissues (*n* = 16), adenocarcinomas with Gleason scores of ≤7 (*n* = 81), adenocarcinomas with Gleason scores of ≥8 (*n* = 19), and SCPC specimens (*n* = 8) from the Duke University School of Medicine. Scale bars, 100 μm. Data were presented as the mean ± SEM. * vs. normal tissues. ***p < 0.001, ****p < 0.0001; by a one-way ANOVA. **C** Mean expression of *PKLR* in human normal (*n* = 28), primary (*n* = 98), and metastatic (*n* = 13) prostate samples in the Taylor PCa dataset. * vs. normal samples. *p < 0.05, **p < 0.01. Significance was determined by a one-way ANOVA. **D** *PKLR* expression in patient samples in the Taylor PCa dataset by pathologic Gleason scores. * vs. GS6. *p < 0.05, **p < 0.01. Significance was determined by a one-way ANOVA. **E** Levels of PKLR mRNA in PCa samples with adenocarcinoma (Adeno) vs. NEPC of the Aggarwal PCa datasets. *p < 0.05; by a one-way ANOVA. **F** GSEA of TCGA PCa dataset revealing significant correlations between higher PKLR expression in prostate tissues with gene signatures representing NED-responsiveness. NES normalized enrichment score, FDR false discovery rate. **G** Kaplan–Meier curve showing survival relative to *PKLR* expression in the Taylor PCa dataset. Patient groups with high PKLR mRNA levels (red line) had lower percentage survival than groups with low PKLR mRNA levels (blue line). The hazard ratio of PKLR low/PKLR high was 0.275; the hazard ratio of PKLR high/PKLR low was 3.632 (by a log-rank test), *p* = 0.036. **H** PKLR, ENO2, CHGA, NKX3-1, and KLK3 protein levels in various PCa cell lines as determined by immunoblotting. **I**, **J** NE markers, stem cell markers, and androgen-responsive gene mRNA levels in LNCaP and PC3 cells following stable transfection with an empty vector (EV)/PKLR-expressing vector (**I**) or non-target control (NC)/PKLR shRNA vector (**J**), by RT-qPCR analysis. * vs. the EV or NC. **K** Immunoblots showing PKLR, ENO2, CHGA, NKX3-1, and KLK3 protein levels in LNCaP and C4-2 cells with charcoal-stripped serum (CSS)-containing medium treatment for 1 week following stable transfection with the NC or PKLR shRNA vector. **L** RT-qPCR showing PKLR, NE markers, stem cell markers, and androgen-responsive gene mRNA levels in parental LNCaP and MDV3100-resistant LNCaP cells. * vs. parental LNCaP; ^#^ vs. the NC; by a two-way ANOVA. Quantification of mRNA is presented as the mean ± SEM from three biological replicates. *p < 0.05, **p < 0.01, ***p < 0.001.

To determine the role of PKLR in PCa progression, we used a tissue microarray (TMA) obtained from the Department of Pathology at Duke University School of Medicine (Durham, NC, USA), which is composed of normal tissues (*n* = 16), adenocarcinomas with a Gleason score of ≤7 (*n* = 81), adenocarcinomas with a Gleason score of ≥8 (*n* = 19), and SCPC specimens (*n* = 8) to determine PKLR expression in different grades of PCa. Interestingly according to immunohistochemical (IHC) staining, we found that high-grade tumors had moderate PKLR expression, and most SCPC cases had high PKLR expression, compared to low-grade tumors and normal prostate tissues (Fig. 1A, B). In studying the clinical relevance of PKLR, we found that prostate tumors from the Taylor PCa dataset [17] with higher PKLR expression were associated with metastasis (Fig. 1C), and a high pathological grade based on the Gleason score (PathGGS) (Fig. 1D). Importantly, levels of PKLR were higher in PCa patients with NEPC compared to patients with adenocarcinomas in Aggarwal datasets [6] (Fig. 1E). Moreover, upregulation of PKLR was positively associated with an NEPC gene signature [18] in The Cancer Genome Atlas (TCGA) PCa database [19] as confirmed by a gene set enrichment analysis (GSEA) (Fig. 1F). Furthermore, Kaplan–Meier analyses showed that patients with tumors exhibiting higher PKLR expression had shorter overall survival than those with low PKLR expression in the Taylor [17] and TCGA [19] PCa datasets (Fig. 1G and Supplementary Fig. S1A). These results supported the hypothesis that PKLR overexpression is involved in malignant progression and may drive the NED of PCa. To understand expression patterns of PKLR in connection with the NED of PCa, we examined protein expression levels of PKLR, NE markers (*ENO2 CHGA*, and androgen-responsive markers (*NKX3-1* and *KLK3*) in a panel of PCa cell lines. We found that the AR-negative PC3 [20] and NE-like NCI-H660 [7] and LASCPC01 [21] cell lines expressed higher PKLR and NE markers and lower androgen-responsive markers than did the AR-positive VCaP, LNCaP, and C4-2 prostate adenocarcinoma cell lines (Fig. 1H). It was shown that NE-like PCa cells are involved in lineage plasticity by stimulating stem cell marker expressions [5]. We found that PKLR modification in PCa cells could alter expressions of NE (*CHGA, CHGB, ENO2*, and *SYP*) markers that are positively associated with stem cell (*NANOG* and *SOX2*) marker expressions and negatively associated with androgen-responsive genes (Fig. 1I and Supplementary Fig. S1B–D). Moreover, combined GSEA results from multiple gene signatures related to neurodevelopment showed positive correlations between PKLR expression and neurodevelopment gene signatures in TCGA PCa dataset according to significant false discovery rates (FDRs) (of <0.25) (GO, KEGG, and Reactome; Supplementary Fig. S1E). These results suggested that PKLR expression may be associated with the NED progression of PCa cells.

It was shown that ADT or more effective AR-targeted therapy may lead to a clinically significant increase in NED-characterized PCa [7]. We found that AR-positive LNCaP and C4-2 cells treated with charcoal-stripped serum (CSS)-containing medium to mimic ADT had higher levels of PKLR, which were positively associated with NE and negatively associated with androgen-responsive markers. However, decreased protein levels of NE markers and increased expressions of androgen-responsive markers were found in PKLR-KD cells regardless of CSS-containing medium treatment (Fig. 1K). Moreover, when LNCaP and C4-2 cells were exposed to long-term treatment with MDV3100 (see also LNCaP-MDVR and C4-2-MDVR cells), a higher level of PKLR was associated with increased NE and stem cell markers and decreased androgen-responsive markers; however, PKLR-KD abolished the alterations of these markers (Fig. 1L and Supplementary Fig. S1F). These results suggest that partial NED after ADT driven by PKLR may be associated with increased stem cell markers.

### Loss of ZBTB10 promotes PKLR-driven NED of PCa after ADT

**Fig. 2:**
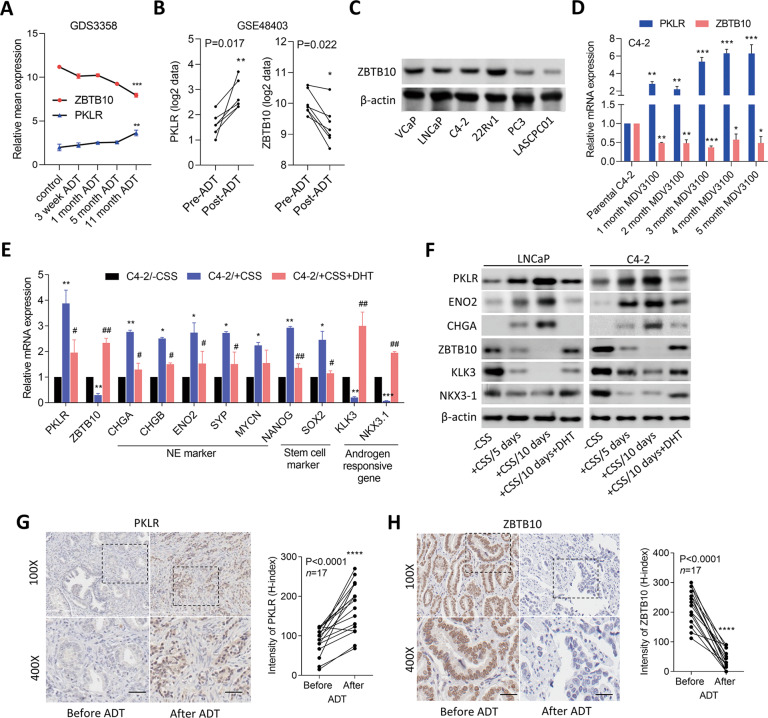
Increased PKLR and decreased ZBTB10 are associated with NED of PCa after ADT. **A** Mean expression of ZBTB10 and PKLR in LNCaP cells from the GDS3358 database during 11 months of ADT. * vs. the control. **p < 0.01, ***p < 0.001; by a one-way ANOVA. **B** PKLR and ZBTB10 expression in patient samples of the GSE48403 dataset showed an increase in PKLR and a decrease in ZBTB10 expression after ADT. *p < 0.05, **p < 0.01; by a one-way ANOVA. **C** Protein levels of ZBTB10 in various PCa cells. **D** PKLR and ZBTB10 mRNA levels in C4-2 cells during 5 months of 20 μM MDV3100/enzalutamide treatment compared to parental C4-2 cells, by RT-qPCR analysis. * vs. parental C4-2; by a one-way ANOVA. **E** RT-qPCR showing PKLR, ZBTB10, NE marker, stem cell marker, and androgen-responsive gene mRNA levels in C4-2 cells following treatment with charcoal-stripped serum (CSS)-containing medium for 48 h, and further treatment with 10 nM dihydrotestosterone (DHT) for 24 h. * vs. -CSS; ^#^ vs. +CSS; by a two-way ANOVA. Quantification of mRNA is presented as the mean ± SEM from three biological replicates. *p < 0.05, **p < 0.01, ***p < 0.001. **F** Immunoblots showing PKLR, ENO2, CHGA, ZBTB10, NKX3-1, and KLK3 protein levels in LNCaP and C4-2 cells following treatment with CSS-containing medium for 5 and 10 days and 10 nM DHT for 24 h. **G**, **H** Representative images of IHC staining and intensity analysis (by an H-score analysis) of PKLR (**G**) and ZBTB10 (**H**) in PCa tissue sections from the same patients before and after ADT. *n* = 17 samples from Taipei Medical University-Wan Fang Hospital. Scale bars, 100 µm. Statistical analysis by two-tailed Student’s *t*-test. ****p < 0.0001.

In order to understand how the suppression of AR signaling upregulates PKLR expression, we analyzed PKLR expression with gene signatures that reflected androgen-responsive genes in the Taylor [17] and TCGA PCa datasets [19]. We found that tissues expressing high levels of PKLR were negatively associated with gene signatures of upregulated androgen responsiveness (Nelson [22], Wang [23], and Hallmark) by a GSEA in both datasets (Supplementary Fig. S2A, B). We focused on AR signaling components that were negatively correlated with PKLR according to GSEA negative rank metric scores in both PCa datasets with PKLR upregulation, and a Venn diagram showed that *MAF, ACSL3, APPBP2*, *PIAS1*, and *ZBTB10* genes overlapped among three androgen-responsiveness signatures (Supplementary Fig. S2C, D). Five candidate genes associated with PKLR were validated by a Pearson correlation analysis, and we found that *ACSL3*, *APPBP2*, *PIAS1*, and *ZBTB10* were significantly negatively correlated with PKLR based on significant *p* values (of <0.0001) in the two PCa datasets (Supplementary Fig. S2E). To determine the effect of ADT on the expressions of these five genes, we examined the mean expression values of these five genes in messenger (m)RNA expression profile data from LNCaP cells cultured during 11 months of androgen deprivation. Results showed that a decrease in ZBTB10 was significantly associated with an increase in PKLR expression compared to other genes (GDS3358, Fig. 2A and Supplementary Fig. S2F). We also observed increased PKLR and decreased ZBTB10 expressions in post-ADT patients compared to pre-ADT patients from mRNA expression profile data (GSE48403, Fig. 2B). Next, we examined protein levels of ZBTB10 in a panel of prostate cell lines and found that AR-negative PC3 and NE-like LASCPC01 cells expressed lower ZBTB10 than AR-positive VCaP, LNCaP, C4-2, and 22Rv1 adenocarcinoma cells (Fig. 2C). We also found an inverse relationship between mRNA levels of ZBTB10 and PKLR in various PCa cells (Supplementary Fig. S3A). To mimic ADT, we treated AR-positive LNCaP and C4-2 cells with MDV3100 for 1–5 months, and results showed that MDV3100-treated cells exhibited significant induction of PKLR and reduction of ZBTB10 compared to parental cells (Fig. 2D and Supplementary Fig. S3B). We also found that upregulation of PKLR was associated with increases in NE and stem cell markers and decreased expressions of ZBTB10 and androgen-responsive genes following CSS-containing medium (to mimic ADT) treatment, whereas the effects of ADT were abolished in the AR ligand dihydrotestosterone (DHT)-rescued cells (Fig. 2E, F and Supplementary Fig. S3C). We further examined the effect of ADT on PKLR and ZBTB10 expressions in clinical samples, which consisted of tissue specimens from 17 PCa patients before and after being treated with ADT collected from Taipei Medical University-Wan Fang Hospital (Taipei, Taiwan). The IHC analysis showed higher levels of PKLR in prostate tumors in patients who received ADT compared to the same patients before ADT; however, ZBTB10 levels were higher in patients before ADT and lower in patients after ADT (Fig. 2G, H). The mean expression correlation of ZBTB10 was validated from the Taylor PCa dataset [17], which showed that ZBTB10 was negatively correlated with NE and stem cell markers and positively correlated with AR-responsive gene expressions according to a Pearson coefficient correlation analysis (Supplementary Fig. S3D). Moreover, tissues expressing high levels of ZBTB10 were negatively associated with upregulated neurodevelopment gene signatures and positively associated with upregulated AR signaling responses as analyzed by GSEAs in TCGA PCa dataset according to significant FDRs (Supplementary Fig. S3E). In summary, these results indicated that inhibition of AR signaling may induce downregulation of ZBTB10 which was correlated with upregulation of PKLR in PCa cells.

### Androgen-activated ZBTB10 directly and negatively regulates *PKLR* expression

**Fig. 3:**
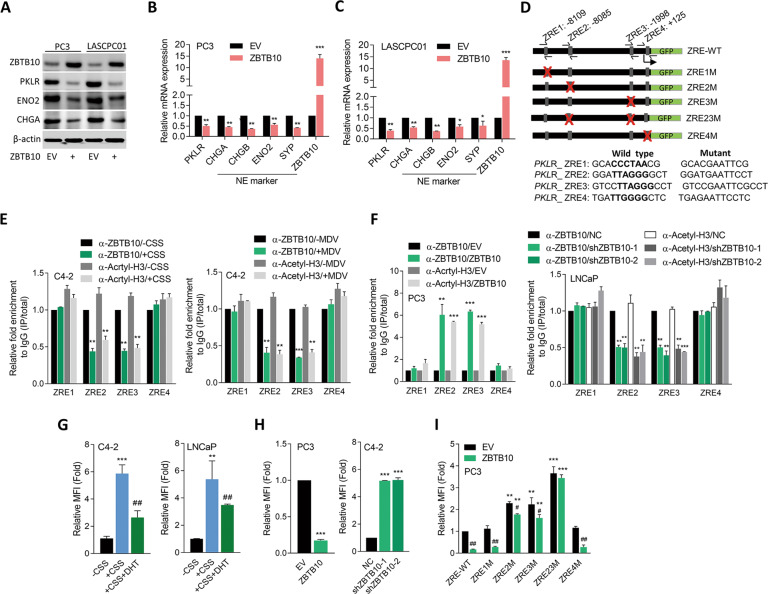
PKLR is downregulated by ZBTB10 in PCa cells. **A** Immunoblots showing ZBTB10, PKLR, ENO2, and CHGA protein levels in PC3 and LASCPC01 cells stably transfected with an empty vector (EV) or ZBTB10 cDNA vector. **B**, **C** RT-qPCR showing PKLR, NE marker, and ZBTB10 mRNA levels in PC3 (**B**) and LASCPC01 (**C**) cells stably transfected with an EV or ZBTB10 cDNA vector. * vs. the EV; by a one-way ANOVA. Quantification of mRNA is presented as the mean ± SEM from three biological replicates. *p < 0.05, **p < 0.01, ***p < 0.001. **D** Schematic of the predicted ZBTB10 response element (ZRE) and various introduced binding site mutants in regulatory sequence reporter constructs of human *PKLR* (GRCh38:1); and sequence diagram of wild-type ZRE (ZRE-WT) and ZRE mutants (ZRE1~ZRE4). **E** ChIP assay showing binding of ZBTB10 and acetyl-H3 to predicted ZREs of the *PKLR* gene regulatory sequence following treatment of C4-2 cells with charcoal-stripped serum (CSS)-containing medium (left) or 10 μM MDV3100 (MDV, right) for 48 h. Sheared chromatin from nuclear extracts was precipitated with antibodies to ZBTB10 and acetyl-H3, and predictive primers (**D**, black arrows) were used to quantify the precipitated DNA by a qPCR. Enrichment of each protein to each site is given as a percentage of the total input and then normalized to IgG. * vs. -CSS (left) or -MDV (right); by a one-way ANOVA. **F** ChIP assay showing binding of ZBTB10 and acetyl-H3 to predicted ZREs of the *PKLR* gene regulatory sequence in PC3 cells following stable transfection with an EV or ZBTB10 cDNA vector (left) or in LNCaP cells following a non-target control (NC) or ZBTB10 shRNA vector (right) transfection. * vs. the EV (left) or NC (right); by a one-way ANOVA. **G** Relative median fluorescent intensity (MFI) of the GFP reporter gene containing ZRE-WT from the *PKLR* regulatory sequence in C4-2 and LNCaP cells following CSS-containing medium treatment for 24 h, and further treatment with 10 nM dihydrotestosterone (DHT) for 24 h. * vs. -CSS; ^#^ vs. +CSS; by a two-way ANOVA. **H** Relative MFI of the GFP reporter gene containing WT-ZRE from the *PKLR* regulatory sequence in PC3 (left) or C4-2 (right) cells following stable transfection with the EV or ZBTB10 cDNA vector (left) or in C4-2 cells following NC or ZBTB10 shRNA vector transfection (right). * vs. the EV (left) or NC (right); by a one-way ANOVA. **I** Relative MFI of the GFP reporter gene containing ZRE-WT or ZREMs from the *PKLR* regulatory sequence in PC3 cells following stable transfection with the EV or ZBTB10 cDNA vector. * vs. ZRE-WT; ^#^ vs. the EV; by a two-way ANOVA. Quantification of the ChIP assay and relative MFI values are presented as the mean ± SEM from three biological replicates. *p < 0.05, **p < 0.01, ***p < 0.001.

ZBTB10 is a transcriptional inhibitor of regulatory genes of the specific protein (SP) family of transcription factors [24], and was shown to play a tumor-suppressive role in ovarian epithelial cancer cells [25]. We hypothesized that ZBTB10 may act as a transcriptional repressor of *PKLR*. We downloaded chromatin immunoprecipitation (ChIP)-sequencing data from GEO (GSE105183) and analyzed it with Genome Brower (Genomics Institute, UCSC). Results showed that ZBTB10 may bind to multiple sites of the *PKLR* gene (Supplementary Fig. S4A). To examine the role of ZBTB10 in mediating expressions of PKLR-associated NE markers, ZBTB10 cDNA was stably transfected into AR-negative PC3 and NE-like LSCPC01 cells and results showed that protein and mRNA levels of PKLR and NE markers were significantly reduced (Fig. 3A–C). Conversely, AR-positive cells with ZBTB10-KD exhibited significantly increased PKLR and NE markers and decreased expressions of AR-responsive markers (Supplementary Fig. S4B–D). We searched for sequences resembling the ZBTB10 response element (ZRE) [26] in *PKLR* regulatory sequences. We found four putative ZREs relative to the transcriptional start site of *PKLR* (Fig. 3D). A ChIP assay was performed and we found that ZRE2 and ZRE3 had significantly decreased ZBTB10-binding ability on *PKLR* after ADT of cells (Fig. 3E). In addition, ZBTB10 overexpression in PC3 cells increased the binding ability of ZBTB10 to ZEB2 and ZEB3 (Fig. 3F, left); however, ZBTB10-KD in LNCP cells reduced the binding ability of ZBTB10 to ZEB2 and ZEB3 (Fig. 3F, right). Reporter assays were performed and we found that CSS-containing medium-treated C4-2 and LNCaP cells showed significantly increased reporter gene activity relative to untreated cells, whereas cells with additional DHT treatment showed decreased reporter gene activity (Fig. 3G). Moreover, ZBTB10 overexpression downregulated reporter activity, while ZBTB10-KD upregulated reporter activity (Fig. 3H), supporting ZBTB10 transcriptional downregulation of *PKLR*. We further used a *PKLR* regulatory sequence-GFP reporter construct in which putative ZRE sites were individually or doubly mutated (Fig. 3D). Results showed that a significant increase in reporter activity was detected at the reporter harboring ZRE2 and ZRE3 mutations (ZRE2M, ZRE3M, and ZRE23M) compared to the ZRE-WT reporter in PC3 cells (Fig. 3I). Moreover, ZBTB10 overexpression downregulated ZRE-WT reporter activity in PC3 cells, while the ZRE2M, ZRE3M, and ZRE23M mutants reduced the suppressive effect of ZBTB10 compared to ZRE1M or ZRE4M reporters, especially the double-mutant ZRE23M (Fig. 3I). These results indicated that ZBTB10 may directly interact with the promoter region of *PKLR*, and downregulation of ZBTB10 through ADT may induce the accumulation of PKLR.

### Loss of ZBTB10 induces PKLR-mediated glucose metabolism and NED in PCa

**Fig. 4:**
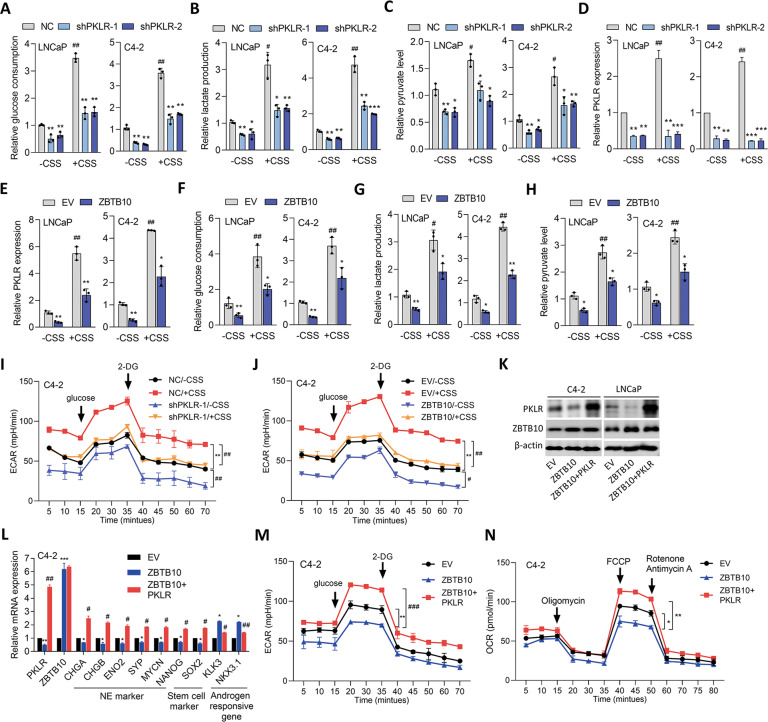
PKLR rescues ZBTB10-decreased glucose metabolism. **A**–**C** Quantification of glucose uptake (**A**), lactate amounts (**B**), and pyruvate levels (**C**) by colorimetric assays of LNCaP and C4-2 cells expressing the non-target control (NC) or PKLR shRNA vector and incubated with charcoal-stripped serum (CSS)-containing medium for 48 h. *n* = 3 per group. * vs. the NC; ^#^ vs. -CSS; by a two-way ANOVA. **D** Relative PKLR mRNA levels in LNCaP and C4-2 cells stably expressing the NC or PKLR shRNA vector. *n* = 3 per group. * vs. the NC; ^#^ vs. -CSS. **p < 0.01, ***p < 0.001; by a two-way ANOVA. **E-G** Quantification of glucose uptake (**E**), lactate amounts (**F**), and pyruvate levels (**G**) by colorimetric assays of LNCaP and C4-2 cells expressing the empty vector (EV) or ZBTB10 cDNA vector and incubated with CSS-containing medium for 48 h. *n* = 3 per group. * vs. the EV; ^#^ vs. -CSS; by a two-way ANOVA. Relative glucose uptake, lactate amounts, and pyruvate levels are presented as the mean ± SEM from three biological replicates. *p < 0.05, **p < 0.01, ***p < 0.001. **H** Relative PKLR mRNA levels in LNCaP and C4-2 cells stably expressing the EV or ZBTB10 cDNA vector. *n* = 3 per group. * vs. the EV; ^#^ vs. -CSS. *p < 0.05, **p < 0.01; by a two-way ANOVA. **I**, **J** Bioenergetics trace from the Seahorse analysis showing values of the extracellular acidification rate (ECAR) in C4-2 cells with expression of the NC or PKLR shRNA vector (**I**) and EV or ZBTB10 cDNA vector (**J**) following treatment with CSS-containing medium for 48 h, and incubated with 12 mM d-glucose and 50 mM 2-deoxyglucose (2-DG). * vs. -CSS; ^#^ vs. the NC (**I**) or EV (**J**); by a two-way ANOVA. **K** PKLR and ZBTB10 protein levels of C4-2 and LNCaP cells stably express the EV, ZBTB10, or ZBTB10+PKLR cDNA vector. **L** Relative PKLR, ZBTB10, NE marker, stem cell marker, and androgen-responsive gene mRNA levels in C4-2 cells expressing the EV, ZBTB10, or ZBTB10 + PKLR cDNA vector. * vs. the EV; ^#^ vs. ZBTB10; by a two-way ANOVA. Quantification of mRNA is presented as the mean ± SEM from three biological replicates. *p < 0.05, **p < 0.01, ***p < 0.001. **M** Bioenergetics trace from the Seahorse analysis showing ECAR values in C4-2 cells with ex*p*ression of the EV, ZBTB10, or ZBTB10+PKLR cDNA vector, and incubated with 12 mM d-glucose and 50 mM 2-DG. **N** Bioenergetics trace from the Seahorse analysis showing oxygen consumption rate (OCR) values in C4-2 cells with stable EV, PKLR, or PKLR+ZBTB10 cDNA vector expression, and incubated with 1 μM oligomycin, 0.75 μM FCCP, and 0.5 μM each of rotenone and antimycin A. * vs. the EV; ^#^ vs. ZBTB10; by a two-way ANOVA. Relative ECAR and OCR values are presented as the mean ± SEM from three biological replicates. *p < 0.05, **p < 0.01, ***p < 0.001.

Previous studies showed that dysregulation of AR signaling can promote glucose metabolism in PCa [27]; however, the mechanism responsible for the abnormalities in glycolytic enzymes caused by ADT-induced metabolic gene and metabolic reprogramming in regulating NEPC development is still unclear. We found that AR-positive LNCaP and C4-2 cells treated ADT showed increased glucose consumption and also higher lactate secretion and pyruvate levels compared to control cells (Fig. 4A–C and Supplementary Fig. S5A). However, PKLR-KD cells showed reductions in these effects, regardless of CSS-containing medium treatment (Fig. 4A–C). Upregulation of PKLR was confirmed in cells treated with CSS-containing medium, but not in CSS-treated PKLR-KD cells (Fig. 4D). Moreover, AR-negative PC3 and NE-like LASCPC01 cells expressing PKLR-KD showed decreased glucose consumption, lactate secretion, and pyruvate levels relative to control cells (Supplementary Fig. S5B). We next analyzed PKLR levels in ZBTB10-overexpressing LNCaP and C4-2 cells in response to ADT. Although ADT increased endogenous PKLR expression, cells overexpressing ZBTB10 exhibited a decrease in PKLR regardless of ADT treatment (Fig. 4E). We also found that ADT could induce higher glucose consumption and lactate and pyruvate levels, while ZBTB10 overexpression reduced these effects (Fig. 4F–H). We further analyzed the extracellular acidification rate (ECAR) in PKLR-KD cells relative to ADT using a Seahorse XFe24 analyzer. Results showed that ECAR values significantly increased in cells responsive to ADT (NC/-CSS vs. NC/ + CSS), while lower ECAR values were found in C4-2 cells with PKLR-KD compared to control cells in the presence or absence of ADT (NC/-CSS vs. shPKLR/-CSS or NC/ + CSS vs. shPKLR/+CSS) (Fig. 4I). Interestingly, PC3 and LASCPC01 cells expressing PKLR-KD had lower ECAR values (Supplementary Fig. S5C, D), supporting the role of PKLR in activating glucose metabolism in AR-negative and NE-like PCa cells. Consistently, we found that cells overexpressing ZBTB10 exhibited reduced ECAR values regardless of ADT conditions (EV/-CSS vs. ZBTB10/-CSS or EV/ + CSS vs. ZBTB10/ + CSS) (Fig. 4). These data suggest that ADT promotes the upregulation of PKLR through the loss of ZBTB10 which may contribute to the mechanism driving glucose metabolism in PCa.

We further rescued PKLR expression into a ZBTB10 cDNA vector (a ZBTB10 cDNA vector without the *ZBTB10* regulatory sequence)-expressing LNCaP and C4-2 cells to evaluate the role of PKLR. Results showed that cells overexpressing ZBTB10 exhibited reduced expressions of endogenous PKLR, while rescued PKLR did not alter the protein levels of exogenous ZBTB10 (Fig. 4K). We also found that overexpressing ZBTB10 can reduce mRNA levels of PKLR, and NE and stem cell markers and upregulation of androgen-responsive genes, whereas rescue of PKLR in ZBTB10-expressing cells was associated with upregulation of NE and stem cell markers and reduction of an androgen-responsive gene (Fig. 4L and Supplementary Fig. S6A). Moreover, glucose consumption and lactate and pyruvate levels were found to be reduced in ZBTB10-overexpressing cells compared to control cells, and rescue of PKLR abolished the effects of ZBTB10 (Supplementary Fig. S6B). We also found that cells with ZBTB10 overexpression had significantly lower ECAR values compared to control cells, whereas upregulation of ECAR values was found in PKLR-recused cells regardless of ZBTB10 overexpression (Fig. 4M and Supplementary Fig. S6C). In addition, we found that cells overexpressing ZBTB10 had lower OCR values, whereas ZBTB10-expressing cells rescued with PKLR had higher OCR values (Fig. 4N and Supplementary Fig. S6D), suggesting a role of PKLR involved in activating oxidative phosphorylation (OXPHOS) in PCa cells. In summary, these data suggested that PKLR may compensate for the ZBTB10-suppressed NED and glucose metabolism of PCa.

### PKLR compensates for the tumor-suppressive effect of ZBTB10

**Fig. 5:**
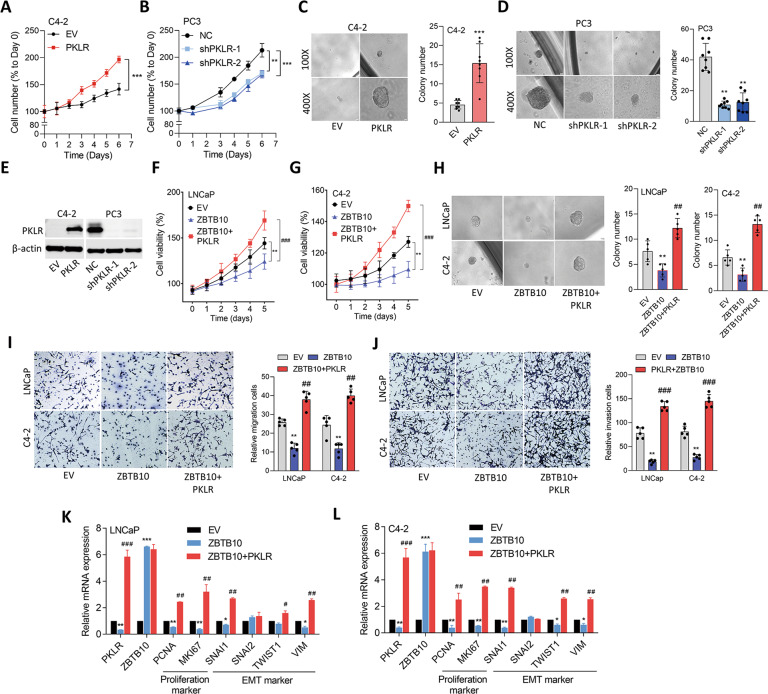
PKLR rescues ZBTB10-suppressed malignancy of PCa. **A**, **B** Proliferation in C4-2 cells stably transfected with an empty vector (EV) or PKLR cDNA vector (**A**) or in PC3 cells with a non-target control (NC) or PKLR shRNA vector transfection (**B**). *n* = 8 per group. * vs. the EV (**A**) or NC (**B**); by a one-way ANOVA. **C**, **D** Sphere formation of C4-2 (**C**) and PC3 (**D**) cells with PKLR overexpression (**C**) or PKLR-knockdown (KD) (**D**), respectively. *n* = 8 per group. * vs. the EV (**C**) or NC (**D**); by a one-way ANOVA. **E** Protein levels of PKLR in C4-2 or PC3 cells with PKLR overexpression or PKLR-KD. **F**, **G** Proliferation of LNCaP and C4-2 cells stably transfected with the EV, ZBTB10, or ZBTB10+PKLR cDNA vector. *n* = 8 per group. * vs. the EV; ^#^ vs. ZBTB10; by a two-way ANOVA. **H** Sphere formation of LNCaP and C4-2 cells stably transfected with the EV, ZBTB10, or ZBTB10+PKLR cDNA vector. *n* = 8 per group. * vs. the EV; ^#^ vs. ZBTB10; by a two-way ANOVA. **I**, **J** Relative migration (**I**) and invasion (**J**) of LNCaP and C4-2 cells stably transfected with the EV, ZBTB10, or ZBTB10 + PKLR cDNA vector. *n* = 5 per group. * vs. the EV; ^#^ vs. ZBTB10; by a two-way ANOVA. Quantification of proliferation, sphere formation, migration, and invasion assays presented as the mean ± SEM from three biological replicates. *p < 0.05, **p < 0.01, ***p < 0.001. **K**, **L** Relative PKLR, ZBTB10, proliferation marker, and EMT marker mRNA levels in LNCaP (**K**) and C4-2 (**L**) cells expressing the EV, PKLR, or ZBTB10 + PKLR cDNA vector. * vs. the EV; ^#^ vs. ZBTB10; by a two-way ANOVA. Quantification of mRNA is presented as the mean ± SEM from three biological replicates. *p < 0.05, **p < 0.01, ***p < 0.001.

Regarding the functional role of PKLR in PCa progression, we found that C4-2 cells overexpressing PKLR exhibited statistically increased growth rates in vitro (Fig. 5A). However, PC3 cells with PKLR-KD exhibited a reduced growth rate compared to cells that carried the control vector (Fig. 5B). Moreover, a three-dimensional sphere-formation assay showed that sphere formation in Matrigel was significantly higher or lower in C4-2 and PC3 cells with PKLR overexpression or PKLR-KD, respectively (Fig. 5C, D). Immunoblotting confirmed the expression of the PKLR protein in cells with PKLR modification (Fig. 5E). Furthermore, we found that ADT-resistant LNCaP cells had higher cell proliferation rates compared to parental cells, whereas cells with PKLR-KD or ZBTB10 overexpression had lower cell proliferation rates regardless of ADT resistance (Supplementary Fig. S7A, B). In addition, we also found that ADT-resistant C4-2 cells had induced numbers of spheres, whereas both PKLR-KD and ZBTB10 overexpression reduced numbers of spheres in parental and ADT-resistant cells (Supplementary Fig. S7C, D). To evaluate the potential tumor-suppressive effect of ZBTB10 in downregulating PKLR-driven PCa progression, we used a stable ZBTB10-expressing clone and compared it to ZBTB10-expressing cells rescued with the PKLR cDNA vector in AR-positive LNCaP and C4-2 cells. An MTT analysis revealed that ZBTB10-overexpressing cells showed a significantly lower growth rate compared to cells carrying the control vector; however, the cell proliferation rate was higher in PKLR-rescued ZBTB10-expressing cells (Fig. 5F, G). Consistently, we found that cells with ZBTB10 expression had decreased sphere formation, while ZBTB10-expressing cells rescued with a PKLR cDNA vector exhibited an increased effect (Fig. 5H). We next monitored cell migration and invasion of ZBTB10-expressing cells compared to PKLR-rescued ZBTB10-expressing cells, and found that cells expressing ZBTB10 showed reduced cell migration and invasion; however, ZBTB10-expressing cells rescued with PKLR exhibited significantly higher cell migration and invasion (Fig. 5I, J). Decreased levels of proliferation markers (*MKI67* and *PCNA*) and epithelial-mesenchymal transition (EMT) markers (*SNAI1, TWIST1*, and *VIM*) were found in ZBTB10-overexpressing cells; whereas higher levels of these markers were found in PKLR-rescued cells regardless of ZBTB10 overexpression (Fig. 5K, L). These data indicated that ZBTB10 may be a tumor-repressive factor that decreases the malignancy and progression of PCa cells; however, PKLR can compensate for the tumor-suppressive role of ZBTB10.

### Targeting PKLR suppresses tumor growth and NED of PCa

**Fig. 6:**
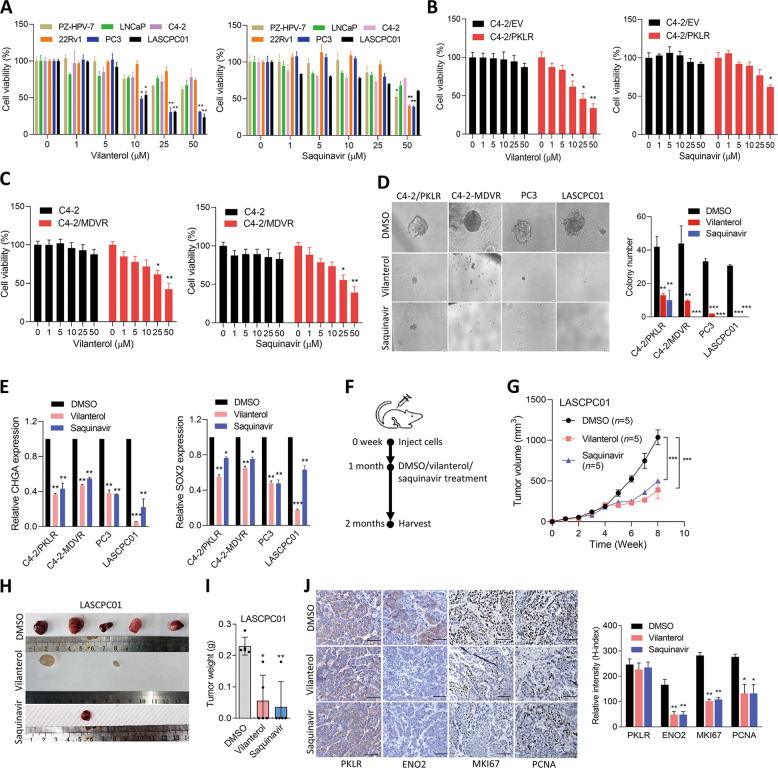
Inhibition of PKLR may be a promising therapeutic strategy of NE-like PCa. **A** Various prostatic cell lines were treated with 0, 1, 5, 10, 25, and 50 μM vilanterol (left) or saquinavir (right) for 24 h, and cell viability was determined by an MTT colorimetric assay. *n* = 8 per group. * vs. the vehicle (0 μM); by a one-way ANOVA. **B** Proliferation analysis of C4-2 cells expressing an empty vector (EV) or PKLR cDNA vector, and treated with 0, 1, 5, 10, 25, and 50 μM vilanterol (left) or saquinavir (right) for 24 h. *n* = 8 per group. * vs. the vehicle (0 μM); by a one-way ANOVA. **C** Proliferation analysis of C4-2 and C4-2-MDVR cells treated with 0, 1, 5, 10, 25, and 50 μM vilanterol (left) or saquinavir (right) for 24 h. *n* = 8 per group. * vs. the vehicle (0 μM); by a one-way ANOVA. **D** Sphere-formation assays of C4-2/PKLR, C4-2-MDVR, PC3, and LASCPC01 cells exposed to the vehicle (DMSO), vilanterol (10 μM), or saquinavir (10 μM) for 1 week. *n* = 8 per group. * vs. DMSO; by a one-way ANOVA. Quantification of cell viability and sphere formation presented as the mean ± SEM from three biological replicates. *p < 0.05, **p < 0.01, ***p < 0.001. **E** Relative CHGA (left) and SOX2 (right) mRNA levels in C4-2/PKLR, C4-2-MDVR, PC3, and LASCPC01 cells with DMSO, 10 μM vilanterol, or 10 μM saquinavir treatment for 24 h. * vs. DMSO; by a one-way ANOVA. Quantification of mRNA is presented as the mean ± SEM from three biological replicates. *p < 0.05, **p < 0.01, ***p < 0.001. **F** Scheme for the treatment of LASCPC01 and C4-2-MDVR xenografts with vilanterol and saquinavir. Male nude mice bearing established LASCPC01 or C4-2-MDVR tumors were treated with DMSO, 25 mg/kg body weight (BW) vilanterol, or 25 mg/kg BW saquinavir starting 1 month after the tumor injection, twice a week for 4 weeks. **G**–**I** Tumor growth analysis of mice subcutaneously inoculated with LASCPC01 cells. Tumor sizes were monitored once a week (**G**), images (**H**), and tumor weights (**I**) were obtained at the end of the experiment (*n* = 5 mice per group). * vs. DMSO. *p < 0.05, **p < 0.01; by a one-way ANOVA. **J** IHC staining and representative intensities of PKLR, ENO2, MKI67, and PCNA in subcutaneous tumors from (**H**). * vs. DMSO. Significance was determined by a two-tailed Student’s *t*-test. *p < 0.05, **p < 0.01.

Since there are no PKLR inhibitors approved for clinical use, we conducted in-house drug testing and analysis to study the promising therapeutic inhibitory effects of approved drugs that target PKLR. In order to find PKLR inhibitors and study the binding mode of PKLR with approved drugs, a molecular docking analysis was performed. Molecular docking was carried out with iGemDock V2.1 [28], using the small-molecule modules downloaded from the ZINC15 database [29], and structural information of the docked PKLR protein (active site, binding site, and allosteric activating site) was obtained from UniProt. When docking was completed, the highest docking simulation of each compound was selected and ranked by the calculated free energy. Putative PKLR inhibitors were further screened based on their cytotoxicity to PCa cells and normal prostate epithelial cells, followed by their ability to inhibit cell growth and sphere formation in vitro. Interestingly, we found that higher doses of vilanterol and saquinavir significantly reduced the viability of AR-negative PC3 and NE-like LASCPC01 cells compared to other drugs, while vilanterol and saquinavir did not significantly decrease the viability of the normal PZ-HPV-7 prostate epithelial cell line or AR-positive LNCaP, C4-2, and 22Rv1 cells according to MTT assays (Fig. 6A and Supplementary Fig. S8A). To test the specificity of targeting PKLR, C4-2 cells with PKLR overexpression (C4-2/PKLR) or MDV3100-resistance (C4-2-MDVR) were treated with these small-molecule drugs, and results showed that C4-2/PKLR or C4-2-MDVR cells treated with vilanterol and saquinavir exhibited significantly reduced growth rates compared to cells treated with other drugs (Fig. 6B, C and Supplementary Fig. S8B–D). Vilanterol and saquinavir did not significantly decrease sphere formation of parental C4-2 and LNCaP cells (Supplementary Fig. S9A). However, C4-2/PKLR, C4-2-MDVR, PC3, and LASCPC01 cells subjected to vilanterol or saquinavir treatment showed reduced sphere formation, compared to cells with DMSO treatment (Fig. 6D). Despite PKLR or ADT inducing glucose metabolism in parental C4-2 and LNCaP cells, both drugs significantly reduced glucose uptake in PKLR-overexpressing or MDV3100-resistant cells; however, neither drug appeared to alter glucose uptake in control cells (Supplementary Fig. S9B, C). Furthermore, C4-2/PKLR, C4-2-MDVR, PC3, and LASCPC01 cells treated with vilanterol and saquinavir exhibited reductions in NE and stem cell markers, but not PKLR (Fig. 6E and Supplementary Fig. S9D), suggesting that vilanterol and saquinavir may be candidate inhibitors of PKLR through directly abolishing functional sites of PKLR. After selection, we identified two candidate PKLR inhibitors, vilanterol and saquinavir, which were further tested in vivo. Mice were subcutaneously injected with LASCPC01 and C4-2-MDVR cells and were further treated with vilanterol or saquinavir after tumor formation (Fig. 6F). We found that mice harboring LASCPC01 or C4-2-MDVR tumor cells with vilanterol or saquinavir treatment exhibited dramatic decreases in tumor growth (Fig. 6G, H and Supplementary Fig. S10A) and lower tumor weights (Fig. 6H, I and Supplementary Fig. S10B), compared to control mice. Interestingly, although we did not see a decrease in PKLR in mice treated with vilanterol or saquinavir, decreased NE (ENO2) and proliferation (MKI67 and PCNA) markers were found in mice after vilanterol or saquinavir treatment, as confirmed by IHC staining of subcutaneous tumors (Fig. 6J and Supplementary Fig. S10C), suggesting that PKLR-targeted therapy might suppress a variety of NED properties as well as growth rates of PCa cells.

### High-grade PCa in patients with reduced ZBTB10 and increased PKLR abundances

**Fig. 7:**
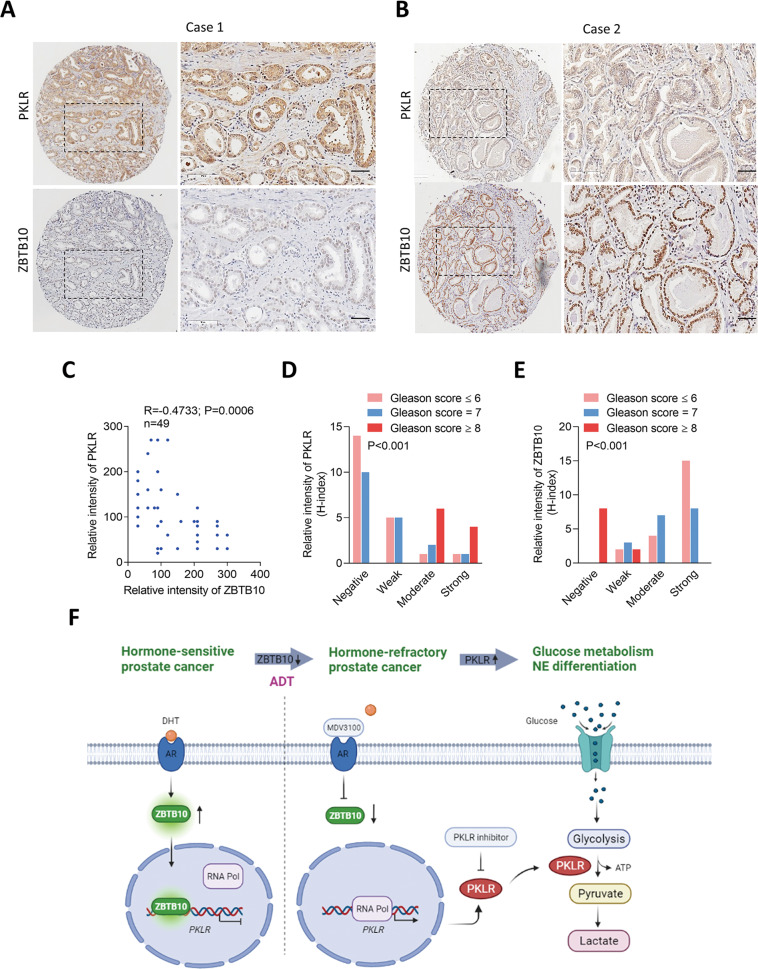
ZBTB10 is negatively correlated with PKLR expression. **A**, **B** IHC staining of PKLR and ZBTB10 on consecutive sections of a PCa TMA (CA4) in two selected cases. Scale bars, 100 μm. **C** Correlation analysis of the intensities of PKLR and ZBTB10 of the PCa TMA (*n* = 49). PKLR expression was negatively associated with ZBTB10-expressing PCa samples. *R*, correlation coefficient; *p*, two-tailed *p* value. Significance was determined by correlation XY analyses in GraphPad Prism. **D**, **E** Tumor grade association analysis using a Chi-squared test of the CA9 PCa TMA. Intensities of PKLR (**D**) and ZBTB10 (**E**) staining were semiquantitatively scored using the H-index as follows: negative, weakly positive, moderately positive, and strongly positive. *p* values were calculated by a Chi-squared test performed using SPSS statistical 18.0 software. *p* < 0.001. **F** Proposed model for ADT-induced PKLR drives hormone-refractory PCa. Hormone-sensitive PCa activates AR signaling by increasing ZBTB10 leading to increased binding to the *PKLR* regulatory sequence and mediation of its transcriptional suppression. ADT induced inactivation of the AR-ZBTB10 pathway, leading to an abundance of PKLR through ZBTB10 loss of function. Overexpression of PKLR may upregulate glucose metabolism and NED progression of PCa cells. Targeting PKLR by potential PKLR inhibitors may reduce the PKLR-driven glycolysis and NED of ADT-resistant PCa.

To study relationships between ZBTB10 and PKLR in clinical tissue samples, we analyzed a PCa TMA (Super Bio Chips #CA4) comprising 49 cases of primary PCa. IHC results showed a reverse correlation between ZBTB10 and PKLR in consecutive tissue sections of prostate tumor samples (Fig. 7A–C). Importantly, high-grade tumors (with Gleason scores of ≥8) had greater abundances of PKLR and lower abundances of nuclear ZBTB10, whereas the reverse was seen in low-grade tumors (with Gleason scores of ≤6) (Fig. 7D, E). Furthermore, since high abundances of PKLR were detected in SCPC samples, we found that the same SCPC samples had lower abundances of ZBTB10 compared to PKLR (Supplementary Fig. S11). These results supported our hypothesis that PKLR abundance is positively correlated with loss of ZBTB10 and is associated with a poor prognosis. In summary, our results demonstrated a regulatory mechanism that inhibition of AR signaling in hormone-sensitive PCa through ADT may cause a reduction in ZBTB10, leading to abundances of PKLR-promoting oncogenic signaling pathway components in a subset of patients with hormone-refractory PCa associated with enhanced glucose metabolism and NE characteristics. Targeting PKLR by drug repurposing with small-molecule PKLR inhibitors may reduce the PKLR-driven glycolysis and NED of ADT-resistant PCa (Fig. 7F).

## Discussion

During the malignant transformation of PCa, the glucose metabolism pathway is frequently abnormal due to the upregulation of genes encoding glycolytic enzymes and pyruvate kinase [8]. PKLR is an isoenzyme of pyruvate kinase encoded by the *PKLR* gene, which is mainly activated in cells during glucose metabolism [10]. We identified a novel signaling pathway that regulates PKLR and provided a specific therapeutic strategy to target ADT-resistant and NE-like PCa cells by pharmacological inhibition of PKLR. We studied key functions of PKLR in regulating glucose metabolism and NED progression in prostate tumors, by illuminating novel functions of PKLR in regulating NED-associated gene expressions. However, a limitation of this study was determining how PKLR regulates gene expression. Our IHC staining results showed that the intensity of nuclear PKLR was increased in high-grade PCa and in PCa samples after ADT resistance, especially a dramatic abundance of nuclear PKLR was seen in aggressive SCPC samples (Figs. 1A, 2G). We hypothesized that PKLR may be translocated into nuclei and act as a transcription factor to enhance its target gene expressions in addition to conducting its kinase function. It was shown that another pyruvate kinase, PKM2, can be translocated into nuclei through activation of the epidermal growth factor receptor (EGFR) pathway [30]. In addition, nuclear PKM2 may also act as a transcription co-activator with hypoxia-inducible factor (HIF)-1α [31, 32]. PKLR might also act similarly to PKM2 and be associated with the hypoxia-HIF-1A pathway in PCa. We found significant alterations in expressions of the AR downstream targets, KLK3 and NKX3-1, in PKLR-modified cells (Fig. 1I, J and Supplementary Fig. S1B–D). These results suggested that modification of KLK3 and NKX3-1 may be due to direct or indirect regulation of nuclear PKLR. We are currently further investigating the mechanism by which PKLR is involved in nuclear translocation during PCa progression.

Our study aimed to illustrate the oncological role of PKLR in PCa progression and its mechanism of upregulation after ADT. ZBTB10 is a novel member of the ZBTB transcription factors, and its biological role has not been fully elucidated. ZBTB10 serves as a transcriptional inhibitor of SP1 transcription factors and participates in inhibiting tumor activities, including tumor growth, chemoresistance, and migration/invasion [33, 34], suggesting that ZBTB10 should be considered a tumor suppressor. In a genome-wide association study focused on gene loci, *ZBTB10* was found to be associated with the sex hormone-binding globulin content [35], which indicates that ZBTB10 might participate in the development of the prostate gland. Through analysis of PCa mRNA expression databases and subsequent in vitro and in vivo experiments, we demonstrated a novel role for ZBTB10, which may be an upstream regulator of PKLR in PCa. Reducing the activity of AR signaling by ADT or pharmacological AR antagonists suppresses the AR, which may downregulate ZBTB10, facilitating PKLR upregulation. This AR/ZBTB10/PKLR axis provides one explanation for the common progression to hormone-refractory PCa after ADT in patients. Our results indicated that loss of ZBTB10 in PCa may be able to support glucose metabolism and NED progression by altering the expression and oncogenic role of PKLR.

We further assessed the potential of targeted PKLR therapy in aggressive PCa treatment. We screened potential PKLR inhibitors from approved drugs and found two drugs, vilanterol and saquinavir, which could be cytotoxic antagonist targets of NE-like and MDV3100-resistant PCa cells. Vilanterol is a β-adrenergic receptor agonist which is usually combined with umeclidinium or fluticasone furoate for treating chronic obstructive pulmonary disease (COPD) and asthma [36]. Our results showed that vilanterol blocked the formation of tumor clusters when treating NE-like and ADT-resistant cells. When administered to mice bearing LASCPC01 cells, vilanterol was found to effectively inhibit tumor growth, suggesting that vilanterol may be a promising drug for treating NE-characterized PCa. Our study is the first report of vilanterol being used for cancer therapy. Saquinavir is a human immunodeficient virus protease inhibitor (HIV-PI) that inhibits the cleavage of polyproteins synthesized from retroviral genomes [37]. Many studies showed that HIV-PIs are also antitumor agents for lung, breast, colon, and prostate tumors [37]. HIV-PIs are used to block internal proteasome activity, causing ER stress or growth factor signal interference, leading to apoptosis of PCa cells [38]. Notably, saquinavir exhibited growth-inhibitory activity against tumor formation of AR-negative PC3 and DU145 cells [39], suggesting the anti-PCa activity of saquinavir. Our cell viability results showed that 10, 25, or 50 μM of vilanterol significantly decreased of the viability of PC3 and LASCPC01 cells by more than 50% compared to the normal PZ-HPV-7 prostate epithelial cell line, whereas 10 or 25 μM of saquinavir displayed no significant difference between PC3/LASCPC01 and normal prostate epithelial cells (Fig. 6A). These results indicated a better cytostatic response of vilanterol compared to saquinavir in PCa.

## Conclusions

Our results showed that an inverse relationship exists between AR/ZBTB10 signaling and PKLR-driven glucose metabolism and NED signaling pathways. Under androgen regulation, ZBTB10 is a critical transcriptional factor for the direct repression of regulatory sequences of *PKLR*, while loss of androgen signaling or inhibition of the AR causes an enhanced malignant phenotype possibly through inactivation of ZBTB10 and activation of PKLR.

## Materials and methods

### Cell culture and reagents

Cell lines used in this study were obtained from American Type Culture Collection (ATCC). The culture medium was distinct for each cell line. LNCaP cells were cultured in Roswell Park Memorial Institute (RPMI) medium 1640 (ThermoFisher, 11875-085), with 5% fetal bovine serum (FBS, US origin, EMD Millipore, TMS-013-BKR). C4-2 and PC3 cells were cultured in RPMI-1640 with 5% heat-inactivated FBS. VCaP cells were cultured in Dulbecco’s modified Eagle medium (DMEM; Corning, 10-013-CV) with 5% FBS. LASCPC01 cells were cultured in HITES medium which is composed of RPMI-1640, 10 nM hydrocortisone (Sigma-Aldrich, H0888), 1× insulin/transferrin/selenite (ThermoFisher, 41400-045), 200 nM β-estradiol (Sigma-Aldrich, E2758), and 5% FBS. NCI-H660 cells were cultured in RPMI-1640 medium supplemented with 1× insulin/transferrin/selenite (ThermoFisher, 41400-045), 10 nM hydrocortisone (Sigma-Aldrich), 10 nM ß-estradiol (Sigma-Aldrich, E2758), 4 mM l-glutamine (Invitrogen), and 5% FBS. The PZ-HPV-7 normal prostate epithelial cell line was cultured in keratinocyte serum-free medium (K-SFM, ThermoFisher, 17005-042) supplemented with 0.05 mg/ml bovine pituitary extract (BPE, ThermoFisher) and 5 ng/ml human recombinant epidermal growth factor (EGF, ThermoFisher). MDV3100/enzalutamide-resistant LNCaP-MDVR and C4-2-MDVR cells were respectively derived from LNCaP and C4-2 cells cultured for 12 months in RPMI-1640 medium with 5% FBS containing 20 μM MDV3100 (Selleckchem, S1250). All cell lines tested negative for mycoplasma contamination. To mimic ADT, cells were cultured in RPMI-1640 medium with 5% CSS (ThermoFisher, 12676-029)-containing medium for 48 h or treated with 10 μM MDV3100 (Selleckchem, S1250) under standard culture conditions for 48 h. The AR ligand was treated with 10 nM DHT (Sigma-Aldrich) for 24 h. The candidate PKLR inhibitors (vilanterol, saquinavir, fosinopril, and salmeterol) were purchased from MedChemExpress (HY-14300, HY-17007, HY-B0382, and HY-14302), and the concentrations of each candidate drug for cell viability were treated with 0, 1, 5, 10, 25, and 50 μM for 24 h.

### Tissue samples

PCa TMA sections from Duke University School of Medicine (Durham, NC, USA) included 16 normal prostatic epithelial samples, 81 adenocarcinomas with a Gleason score of ≤7, 19 adenocarcinomas with a Gleason score of ≥8, and eight SCPC samples, and their use was approved by the Duke University School of Medicine Institutional Review Board (protocol ID: Pro00070193). PCa TMA sections comprising PKLR and ZBTB10 in 49 cases of primary prostate adenocarcinoma were purchased from Super Bio Chips (CA4). Tumor samples of 17 PCa patients before and after ADT treatment were collected from Taipei Medical University-associated Wan Fang Hospital following a protocol based on the *Declaration of Helsinki*, and the study protocol was approved by the Taipei Medical University Joint Institutional Review Board (approval ID: N202001020).

### IHC staining

For IHC staining, slices were deparaffinized and rehydrated, the antigen was retrieved using heat, and slices were stained with the first antibody (PKLR, 1:100, Abcam, ab125697; ZBTB10, 1:200, Invitrogen, PA5-54448; enolase 2 (ENO2), 1:100, Abcam, ab218388; a marker of proliferation KI-67 (MKI67), 1:200, Abcam, ab15580; and proliferating cell nuclear antigen (PCNA), 1:200, Abcam, ab29) at 4 °C. Then, slices were stained with the second antibody (horseradish peroxidase (HRP) anti-rabbit or HRP anti-mouse, Dako, E0432 or E0433) after proper washing with Tris-buffered saline (TBS) buffer with 0.1% Triton X-100, conjugated with avidin, and colorized with the 3,3′-diaminobenzidine (DAB, Dako, K3468) reagent. Then, slices were dehydrated and mounted with glycerol, and a snapshot was taken with a phase-contrast microscope (Olympus IX73). The intensity of the target gene in a slice was diagnosed by a pathologist, and the intensity was defined as 0 (negative), 1+ (weakly positive), 2+ (moderately positive), or 3+ (strongly positive) for H-index calculation according to the following formula [40]: ${{{\mathrm{H}}}}\,{{{\mathrm{index}}}} = [1 \times ({{{\mathrm{\% }}}}\,{{{\mathrm{cells}}}}\,{{{\mathrm{of}}}}\,1 + )] + [2 \times ({{{\mathrm{\% }}}}\,{{{\mathrm{cells}}}}\,{{{\mathrm{of}}}}\,2 + )] + [3 \times ({{{\mathrm{\% }}}}\,{{{\mathrm{cells}}}}\,{{{\mathrm{of}}}}\,3 + )].$

### Proliferation assay

Cell proliferation was tested using the 3-(4,5-dimethylthiazol-2-yl)-2,5-diphenyltetrazolium bromide (MTT) assay (Santa Cruz, sc-359848), in which 5 × 10^3^ cells per well were seeded in 96-well plates and cultured overnight until confluent. From the next day (day 0), one plate was picked to measure cell numbers, and the remaining ones were continued culturing under standard culture conditions. MTT solution (1 mg/mL at 100 μL/well) was added to the test plates (to a final concentration of 500 μg/mL) and incubated an additional 4 h under standard culture conditions. Later, the MTT-containing medium was replaced with 50 μL/well dimethyl sulfoxide (DMSO) and incubated for 10 min in the dark to completely dissolve the formazan crystals. The optical density at 570 nm (OD_570_) was measured in each well with a microplate reader (BioTek). Relative cell numbers are presented as the OD_570_ ratio normalized to day 0.

### Three-dimensional sphere-formation assay

A protocol of three-dimensional growth in Matrigel was followed from our previous study with slight modification [41]. In brief, 500 cells/well of desired cells were suspended in 50 μL of complete medium followed by mixing with an aliquot of standard Matrigel matrix (Corning, 354234). Then, the mixture was loaded into the bottom edge of a 24-well plate and incubated overnight for aggregation. The next day (day 0), 2 mL of culture medium was loaded into the wells and cultured for 1 week. To evaluate the cytotoxicity of candidate PKLR inhibitors, LNCaP, C4-2, C4-2/PKLR, C4-2-MDVR, PC3, and LASCPC01 cells were treated with 10 μM of vilanterol or saquinavir for 1 week. Finally, tumorspheres in each well were observed, a snapshot was taken with phase-contrast microscopy (Olympus), and the spheres were counted.

### Tumorigenicity assays in mice

The protocol of the in vivo tumorigenicity assay was based on *Guidelines for Care and Use of Laboratory Animals* from the Council of Agriculture, Executive Yuan, Taiwan and was approved by the Taipei Medical University Institutional Animal Care and Use Committee (approval ID: LAC-2020-0032). Six-week-old CAnN.Cg-*Foxn1*^*nu*^/CrlNarl male mice were purchased from the National Laboratory Animal Center (NLAC, Taipei, Taiwan). Mice were randomized into three groups and subcutaneously injected with 10^6^ cells/site (suspended in 100 μL of an aliquot mixture of Matrigel matrix and culture medium) of LASCPC01 and C4-2-MDVR cells in the right side of the flank, and the tumor diameter and mouse weight were monitored once a week. One month after injecting the cells, the mice were intraperitoneally injected with DMSO, vilanterol (25 mg/kg), or saquinavir (25 mg/kg) twice a week for 4 weeks under double-blind conditions. The tumor diameter was converted into a volume according to the following formula [42]: *V* = 0.5236 × *H* × *W* × *L*; where V, H, W, and L are the volume, height, width, and length, respectively.

### ChIP assay

A ChIP assay was carried out using the EZ-Magna ChIP^TM^ IP kit A (Sigma-Aldrich, 17-10086) following the protocol recommended in the manual. After treatment, 10^6^ cells were fixed with 1% paraformaldehyde/complete medium for 10 min followed by termination of fixing by incubating with 125 mM glycine buffer for 5 min. Fixed cells were washed with PBS with proteinase and phosphatase inhibitors in a freezer. Then, cells were scraped under PBS buffer, and the debris was collected. Chromatin within the debris was released using the lysis buffer in the kit and was disrupted into 150-bp pieces by sonication (Qsonica). Chromatin–protein complexes were labeled with 10 ng of an anti-ZBTB10 antibody (Abcam, ab117786), anti-acetyl-histone H3 antibody (positive control, Novus, NB300-221), or normal rabbit immunoglobulin G (IgG) (negative control, Santa Cruz, sc-2027) followed by enrichment using protein A-coated magnetic beads. Chromatin was released from the complexes by proteinase K (Sigma-Aldrich, 124568) following heat inactivation, and were identified by a reverse-transcription quantitative polymerase chain reaction (RT-qPCR). ChIP antibodies and qPCR primers are listed in Supplementary Table S4. For the ChIP-sequencing analysis, ChIP-sequencing data were downloaded from Gene Expression Omnibus (GEO) (GSE105183) and analyzed by Genome Brower (Genomics Institute, UCSC, CA, USA).

### Promoter reporter assay

ZBTB10 response elements (ZREs) are located upstream of human *PKLR* on chromosome 1: 155291131 (ZRE1: -8109), 155291788 (ZRE2: -8085), 155297844 (ZRE3: -1998), and 155299967 (ZRE4: +125) at GRCh38. These regulatory sequences with response-element green fluorescence protein (GFP) reporter vectors were constructed using the pGreenFire1-ISRE Lentivector (System Biosciences, TR016PA-P). Cells (5 × 10^4^ cells/well) in 12-well plates were transiently transfected with 1 µg of the wild-type (WT)- and mutant (M)-*PKLR*-GFP reporter containing ZREs using the X-tremeGENE™ HP DNA transfection reagent. To mimic ADT, transfected C4-2 and LNCaP cells were treated with CSS-containing medium or 10 μM MDV3100 for 48 h. The WT-*PKLR*-GFP and M-*PKLR*-GFP reporters were co-transfected with an empty vector (EV) or ZBTB10-expressing vector in PC3 cells or co-transfected with a non-target control (NC) or a ZBTB10 short hairpin (sh)RNA vector in LNCaP or C4-2 cells. The promoter function was analyzed using fluorescence-activated cell sorting (FACS, BD Biosciences), and relative median fluorescent intensity (MFI) values were measured for GFP by FACS using FACSDiva software (BD Biosciences) and normalized to the value of the vehicle. Three independent experiments were run with triplicate samples.

### Colorimetric assay

Colorimetric assay kits for glucose consumption, lactate production, and intracellular pyruvate contents were purchased from Abcam (ab136955, ab65331, and ab65342, respectively), and the assay protocol followed the description in the manual. Briefly, 4 × 10^6^ cells with indicated treatments were inoculated into 10-cm cultural dishes and cultured overnight for cell attachment. Then, attached cells were cultured for an additional 48 h in a renewed medium as indicated. When culturing was complete, a medium from each treatment was collected for glucose, lactate, and pyruvate content assays. The remaining cells were detached with trypsin, harvested, washed with PBS, and used to determine the intracellular pyruvate content.

### ECAR analysis

A glycolytic rate assessment was performed with a Seahorse XFe24 analyzer (Seahorse Bioscience) coupled with a glycolytic rate assay kit (Seahorse Bioscience, 103344-100). In total, 5 × 10^4^ cells/well of desired cells (at about 90% confluence) were inoculated into a Seahorse analyzer-specific culture plate (Seahorse Bioscience) and incubated overnight until confluent. The next day, cells were washed with pre-warmed serum-free, phenol-red-free, and glucose-free medium followed by culturing with serum-free, phenol-red-free, and glucose-free medium supplemented with 4-(2-hydroxyethyl)-1-piperazineethanesulfonic acid (HEPES), 2 mM glutamine, and 1 mM sodium pyruvate. The ECAR analysis was used to obtain bioenergetic parameters in C4-2 or LNCaP cells expressing the NC or PKLR shRNA vector or the EV, ZBTB10 complementary (c)DNA vector following CSS-containing medium treatment and treated with the sequential addition of 12 mM d-glucose and 50 mM 2-deoxyglucose (2-DG), a competitive inhibitor of glucose.

### Oxygen consumption rate (OCR) analysis

OCR measurements were obtained over time (min) using a Seahorse XF24 extracellular flux analyzer (Seahorse Bioscience) coupled with a Mito stress assay kit (Seahorse Bioscience, 103015-100). In total, 5 × 10^4^ cells/well of desired cells (at about 90% confluence) were inoculated into a Seahorse analyzer-specific culture plate (Seahorse Bioscience) and incubated overnight until confluent. A mitochondrial stress test was used to obtain bioenergetic parameters in C4-2 cells expressing the EV or ZBTB10 or ZBTB10 + PKLR cDNA vector following the sequential addition of inhibitors of mitochondrial function: 1 μM oligomycin, 0.75 μM carbonyl cyanide-ptrifluoromethoxyphenylhydrazone (FCCP), and a combination of rotenone and antimycin A (at 0.5 μM each).

### Migration and invasion assays

Migration and invasion assays followed protocols in the literature with slight modifications [43]. For migration, totals of 3 × 10^3^ cells/well were suspended in serum-free medium and inoculated in a 24-well Boyden chamber (8-μm pore size), and the chamber was put into a 24-well culture plate. For the invasion assay, totals of 2 × 10^3^ cells/well were incubated in the same Boyden chamber pre-coated with 200 μg/mL Matrigel matrix (Corning, 354234). Matrigel-coated transwell dishes were prepared by adding 200 μl of CSS-containing medium diluted with Matrigel. The lower chamber was filled with 600 μl of the complete medium outside the chamber. The plates were incubated for 24 h for migration and 8 h for an invasion before staining the chamber with a 0.5% crystal violet/methanol/PBS solution for 20 min. After washing with PBS, cells in the chamber which had not invaded were removed with a cotton swab, and invaded cells on the underside of the membrane were counted and quantified in five medium-power fields for triplicate replicates.

### Statistical analysis

All experiments were independently carried out at least three times. Results of each experiment were collected and plotted using the standard error of the mean (SEM) with GraphPad Prism V8.0 (GraphPad Software). Statistical differences between individual groups were determined by a one- or two-way analysis of variance (ANOVA) followed by Bonferroni’s post hoc test. For comparison of IHC staining of ADT samples, paired two-tailed Student’s *t*-tests were performed. For tumor grade association analysis of IHC staining of the CA9 TMA, *p* values were calculated by a Chi-squared test performed with SPSS statistical software vers. 18.0 (IBM SPSS). For correlations between IHC staining results of PKLR and ZBTB10, significance was determined by correlation XY analyses in GraphPad Prism. A log-rank test was used for the survival curve analysis in the Taylor [17] PCa clinical datasets with GraphPad Prism. The method for determining cutoffs was pre-decided by half of the number of patients. Tumors were mean-stratified by PKLR expression, and the overall survival of patients was determined in each group.

## Supplementary information

Supplementary Materials and Methods

Supplementary Fig. S1

Supplementary Fig. S2

Supplementary Fig. S3

Supplementary Fig. S4

Supplementary Fig. S5

Supplementary Fig. S6

Supplementary Fig. S7

Supplementary Fig. S8

Supplementary Fig. S9

Supplementary Fig. S10

Supplementary Fig. S11

Original Data File

reproducibility checklist

## Data Availability

The reagents used in the current study are available from the corresponding author on reasonable request.
