# Supplementary material for: Pyruvate kinase L/R links metabolism dysfunction to neuroendocrine differentiation of prostate cancer by ZBTB10 deficiency
Source: Cell Death Dis. 2022 Mar 19;13(3):252. doi: 10.1038/s41419-022-04694-z (PMC8934352; doi:10.1038/s41419-022-04694-z)
Supplement: Supplementary file 1 — Supplementary Materials and Methods [file 41419_2022_4694_MOESM1_ESM.docx]

**Supplemental File 1**

**Supplementary Materials and Methods**

**Lentiviral preparation for gene modification**

Gene overexpression and knockdown (KD) were performed with a pseudotype lentiviral transfection system ^1^. For gene overexpression, full-length complementary (c)DNA of the *PKLR* or *ZBTB10* gene was constructed in a pCDH-CMV-MCS-EF1-Puro (System Bioscience) or pCDH-CMV-MCS-pCDH-EF1α-MCS-IRES-Neo lentiviral vector (for ZBTB10 rescue only, System Bioscience) and co-transfected with psPAX2 (Addgene plasmid, #12260) and pCMV-VSV-G (Addgene plasmid, #8454) into 293T cells using X-tremeGENE™ HP DNA Transfection Reagent (Roche). 293T cells were cultured in DMEM with 5% heat-inactivated FBS. To all medium was added 1× penicillin/streptomycin (ThermoFisher, 15070-063), 1× non-essential amino acids (NEAAs, ThermoFisher, 11140-050), 1 mM sodium pyruvate (ThermoFisher, 11360-070), and 1× GlutaMAX (ThermoFisher, 35050-061). Later, transfection medium was replaced as the culture medium and cultured for an additional 48 h. The virus-enriched supernatant was collected, passed through a 0.45-μm filter, and stored at -80 °C until being used. For gene overexpression, pseudotyped lentiviral vectors, pCDH-CMV-MCS-EF1-Puro or pCDH-CMV-MCS-EF1-Neo plasmids (System Biosciences), were used for PKLR and ZBTB10 overexpression. For gene KD, the non-target control (NC) or pKLO.1 vector with short hairpin (sh)RNA for *PKLR* or *ZBTB10* was purchased from the RNAi Core Facility (Academia Sinica, Taipei, Taiwan). Promoter reporters were constructed using the pGreenFire1-ISRE Lentivector (System Biosciences), and a site-directed mutagenesis system kit (ThermoFisher) was used for response element mutations. All plasmids were isolated with a Plasmid Midi Kit (Qiagen), and packaged as pseudotype lentiviruses using the same protocol with an overexpressing plasmid. All primers used to generate these constructs are listed in Supplementary Table S1. All constructs were verified by a DNA sequence analysis.

**Clinical dataset analyses**

To compare PKLR and ZBTB10 expression levels with prostate cancer (PCa) progression, we used messenger (m)RNA expression data from the Taylor ^2^, The Cancer Genome Atlas (TCGA) ^3^, Beltran ^4^, and Aggarwal ^5^ human PCa datasets. Expression data were log2-normalized. Gene set enrichment analysis (GSEA) software and gene signatures of androgen-upregulated (Gene Ontology (GO), Pathway Interaction Database (PID), Wang ^6^, Nelson ^7^, and Hallmark) and neuronal developmental-responsive (GO, Kyoto Encyclopedia of Genes and Genomes (KEGG), and Reactome) datasets were downloaded from the Broad Institute ^8^ and used to determine correlations with PKLR and ZBTB10 levels. Normalized enrichment scores (NESs) and false discovery rates (FDRs) were calculated using the GSEA program. Cutoff values used to identify “PKLR high”, or “ZBTB10 high” patients were pre-determined by half the number of patients from the GSEAs. Tumors were mean-stratified by PKLR and ZBTB10 expressions, and the mean expression of each gene was determined in each group. Pearson coefficient correlations among mRNA levels of PKLR, ZBTB10, MAF, ACSL3, APPBP2, PIAS1, neuroendocrine (NE), and stem cell markers, and androgen-responsive genes were validated using the Taylor ^2^ and TCGA ^3^ PCa datasets. For overall survival, the study used the Taylor clinical PCa dataset ^2^, which was accessed from the Memorial-Sloan Kettering Cancer Center (MSKCC) Cancer Genomics data portal (http://cbio.mskcc.org/cancergenomics/prostate/data/), from which we downloaded clinical and publicly available gene expression data on 98 primary and 13 metastatic PCa samples. In total, 110 patients (98 primary and 13 metastatic PCa samples) were divided into two groups according to their mean PCK1 expression: high PKLR expression (PKLR high, *n*=56) and low PKLR expression (PKLR low, *n*=55). A log-rank test was used for the survival curve analysis, and the hazard ratios of PKLR low/PKLR high and PKLR high/PKLR low were determined in each group.

**Reverse-transcription quantitative polymerase chain reaction (RT-qPCR)**

Total mRNA of appropriately treated cells was isolated with an RNeasy Midi Kit (Qiagen) and quantified using a Nanodrop 2000 analyzer (ThermoFisher). One microgram of total RNA was reverse-transcribed into cDNA using an iScript^TM^ cDNA synthesis kit (Bio-Rad). One microliter of cDNA product was mixed with 1 μL primer mix (at a final concentration of 500 nM), 10 μL iTaq universal SYBR green supermix (Bio-Rad), and 8 μL RNase-free water followed by a qPCR analysis using the StepOnePlus real-time PCR system (ThermoFisher). Reactions for all primer pairs were performed using a thermocycler at an initial temperature of 95 °C for 10 min, followed by 40 cycles of 95 °C for 15 s and 60 °C for 1 min. Normalization was performed by measuring human glyceraldehyde-3-phosphate dehydrogenase (*GAPDH*) expression, which was run in triplicate. All primers used for the PCR are listed in Supplementary Table S2.

**Western blot analysis**

At least 10^6^ appropriately treated cells were harvested, washed with phosphate-buffered saline (PBS), and lysed with radioimmunoprecipitation assay (RIPA, Bio-Rad) buffer containing a proteinase and phosphatase inhibitor cocktail (Sigma-Aldrich, PPC1010) in a freezer. Supernatants were collected by centrifugation at 16,000 rpm for 30 min at 4 °C and quantified using the Bradford assay (Bio-Rad, 5000006). Twenty micrograms of total protein was mixed with protein loading buffer, boiled at 92 °C for 10 min, and separated using sodium dodecylsulfate polyacrylamide gel electrophoresis (SDS-PAGE). Separated proteins were transferred to polyvinylidene difluoride (PVDF) or nitrocellulose membranes, blocked by 2.5% bovine serum albumin (BSA), and stained with the appropriate 1^st^ antibody overnight at 4 °C. Afterwards, the membrane was washed with TBS buffer containing 0.1% Tween-20 and stained with the 2^nd^ antibody. All antibodies used are listed in Supplementary Table S3. Finally, the membrane was soaked in horseradish peroxidase (HRP) substrate (EMD Millipore, WBULS0100), and a snapshot was taken with Cytiva Imager 600 (Amersham PLC).

**In-house drug screening**

Drug screening was performed through molecular docking using iGemDock V2.1 ^9^. According to the protein information from Uniprot, PKLR has three potential docking sites, including substrate binding sites (R ^116^, K ^313^, G ^338^, D ^339^, and T ^371^), a transition state stabilization site (K^313^), and allosteric activating sites (W ^525^ and R ^532^) ^10, 11^. The protein module of PKLR (PDB ID 4IP7) was downloaded from the RCSB Protein Data Bank ^12^ , and docking site selection was performed using the Swiss-PDB viewer, which contains amino acids within 6 Å ^13^. The highest docking simulation of each compound was selected, and ranked according to the calculated free energy. From the list of the candidate compounds vilanterol, saquinavir, fosinopril, and salmeterol were selected as putative PKLR inhibitors.

**Supplementary Figure Legends**

**Supplementary Fig. S1.** **PKLR is inactivated by AR signaling and negatively associated with ZBTB10 expression.** **A** Kaplan-Meier curve showing survival relative to PKLR expression in the TCGA PCa dataset. Patient groups with high PKLR mRNA levels (pink line) had lower percentage survival than groups with low PKLR mRNA levels (blue line). Significance was determined by a log rank test. *p*=0.045. **B, C** NE marker, stem cell marker, and androgen-responsive gene mRNA levels in C4-2 and LASCPC01 cells following stable transfection with an empty vector (EV) and PKLR-expressing vector (**B**) or a non-target control (NC) and PKLR shRNA vector (**C**), respectively, were determined by an RT-qPCR analysis. * vs. the EV or NC. **D** Immunoblots showing PKLR, ENO2, CHGA, NKX3-1, and KLK3 protein levels in LNCaP and C4-2 cells stably transfected with the EV or PKLR-expressing vector (left) or in PC3 and LASCPC01 cells stably transfected with the NC or PKLR shRNA vector (right). **E** GSEAs of TCGA PCa dataset show that higher PKLR expression of PCa tissues was significantly positively associated with neurodevelopment gene signatures (GO, KEGG, and Reactome). NES, normalized enrichment score; FDR, false discovery rate. **F** RT-qPCR showing PKLR, NE marker, stem cell marker, and androgen-responsive gene mRNA levels in parental C4-2 and MDV3100-resistant C4-2 cells. * vs. parental C4-2; ^#^ vs. the NC; by a two-way ANOVA. Quantification of mRNA is presented as the mean ± SEM from three biological replicates. * *p*<0.05, ** *p*<0.01, *** *p*<0.001.

**Supplementary Fig. S2.** **ZBTB10 may act as a mediator of *PKLR*. A, B** GSEAs of the Taylor (**A**) and TCGA (**B**) PCa datasets shows that higher PKLR expression of PCa tissues was significantly negatively associated with three androgen-responsive (Nelson, Wang, and Hallmark) gene signatures. NES, normalized enrichment score; FDR, false discovery rate. **C, D** Venn diagram showing five genes overlapping among three androgen-responsive gene signatures (Wang, Nelson, and Hallmark) of the GSEA results of the Taylor (**A**) and TCGA (**B**) PCa datasets based on negative ranking metric scores. **E** List of genes negatively correlated with PKLR mRNA levels among the gene signatures of three overlapping androgen response signatures in the Taylor and TCGA PCa datasets. **F** Mean expression of MAF, ACSL3, APPBP2, and PIAS1 in LNCaP cells from the GDS3358 database during 11 months of ADT. * vs. the control; by a one-way ANOVA.

**Supplementary Fig. S3.** **ADT causes loss of ZBTB10, which is negatively correlated with PKLR. A** RT-qPCR showing mRNA levels of PKLR and ZBTB10 in various PCa cell lines. * vs. PKLR; by a one-way ANOVA. **B** PKLR and ZBTB10 mRNA levels in LNCaP cells during 4 months of 20-μM MDV3100/enzalutamide treatment, by a RT-qPCR analysis. * vs. parental LNCaP; by a one-way ANOVA. **C** RT-qPCR showing PKLR, ZBTB10, NE marker, stem cell marker, and androgen-responsive gene mRNA levels in LNCaP cells following treatment with charcoal-stripped serum (CSS)-containing medium for 48 h, and further treated with 10 nM dihydrotestosterone (DHT) for 24 h. * vs. -CSS; ^#^ vs. +CSS; by a two-way ANOVA. Quantification of mRNA is presented as the mean ± SEM from three biological replicates. * *p*<0.05, ** *p*<0.01, *** *p*<0.001. **D** Correlation analysis of ZBTB10 among NE marker, stem cell marker, and androgen-responsive gene mRNA levels in prostate tissue samples from the Taylor PCa datasets. Correlation coefficients (*R^2^*) and *p* values were determined by correlation XY analyses in GraphPad Prism. ** *p*<0.01, *** *p*<0.001, **** *p*<0.0001. **E** GSEAs of TCGA PCa dataset showing enrichment of ZBTB10 expression among gene sets, the expression levels of which were negatively associated with neuron development responses (red) and positively associated with androgen signaling responses (blue). NES, normalized enrichment score; FDR, false discovery rate.

**Supplementary Fig. S4.** **Loss of ZBTB10 upregulates *PKLR* expression. A** ChIP-sequencing analysis of detected DNA-binding sites for ZBTB10 of the *PKLR* gene in HEK293 cells labeled as black boxes in the tracks. ChIP-sequencing data were downloaded from Gene Expression Omnibus (GEO) (GSE105183) and analyzed by Genome Brower (Genomics Institute). **B** Immunoblots showing ZBTB10, PKLR, ENO2, CHGA, NKX3-1, and KLK3 protein levels in LNCaP and C4-2 cells expressing the non-target control (NC) or ZBTB10 shRNA vector. **C, D** Relative PKLR, NE marker, androgen responsive gene, and ZBTB10 mRNA levels in LNCaP (**C**) and C4-2 (**D**) cells expressing the NC or ZBTB10 shRNA vector. * vs. the NC; by a one-way ANOVA. Quantification of mRNA is presented as the mean ± SEM from three biological replicates. * *p*<0.05, ** *p*<0.01, *** *p*<0.001.

**Supplementary Fig. S5. Loss of PKLR reduces glucose metabolism of PCa. A** Quantification of glucose uptake, lactate amounts, and pyruvate contents by colorimetric assays of LNCaP and C4-2 cells following 10 μM MDV3100 treatment for 48 h. * vs. -MDV3100; by a one-way ANOVA. **B** Quantification of glucose uptake, lactate amounts, and pyruvate contents by colorimetric assays of PC3 and LASCPC01 cells expressing the non-target control (NC) or PKLR shRNA vector. * vs. the NC; by a one-way ANOVA. Relative glucose uptake, lactate amounts, and pyruvate levels are presented as the mean ± SEM from three biological replicates. * *p*<0.05, ** *p*<0.01, *** *p*<0.001. **C, D** Bioenergetics trace from the Seahorse analysis showing values of the extracellular acidification rate (ECAR) in PC3 (**C**) and LASCPC01 (**D**) cells with the NC or PKLR shRNA vector expression, and incubated with 12 mM D-glucose and 50 mM 2-deoxyglucose (2-DG). Relative ECAR values are presented as the mean ± SEM from three biological replicates. Significance was determined by a two-way ANOVA. ** *p*<0.01.

**Supplementary Fig. S6. PKLR** **reverses** **ZBTB10-suppressing NED and glucose metabolism. A** Relative PKLR, ZBTB10, NE marker, stem cell marker, and androgen-responsive gene mRNA levels in LNCaP cells expressing an empty vector (EV), or ZBTB10 or ZBTB10 + PKLR cDNA vector. * vs. the EV; # vs. ZBTB10; by a two-way ANOVA. Quantification of mRNA is presented as the mean ± SEM from three biological replicates. * *p*<0.05, ** *p*<0.01, *** *p*<0.001. **B** Quantification of glucose uptake, lactate amounts, and pyruvate contents by colorimetric assays of LNCaP and C4-2 cells expressing the EV, or ZBTB10 or ZBTB10 + PKLR cDNA vector. * vs. the EV; ^#^ vs. ZBTB10; by a two-way ANOVA. Relative glucose uptake, lactate amounts, and pyruvate levels are presented as the mean ± SEM from three biological replicates. * *p*<0.05, ** *p*<0.01, *** *p*<0.001. **C** Bioenergetics trace from the Seahorse analysis showing values of the extracellular acidification rate (ECAR) in LNCaP cells with the EV, ZBTB10, or ZBTB10 + PKLR cDNA vector expression, and incubated with 12 mM D-glucose and 50 mM 2-deoxyglucose (2-DG). * vs. the EV; ^#^ vs. ZBTB10; by a two-way ANOVA. **D** Bioenergetics trace from the Seahorse analysis showing values of the oxygen consumption rate (OCR) in LNCaP cells with stable EV, PKLR, or PKLR + ZBTB10 cDNA vector expression, and incubated with 1 μM oligomycin, 0.75 μM FCCP, and 0.5 μM each of rotenone and antimycin A. * vs. EV; ^#^ vs. ZBTB10; by a two-way ANOVA. Relative ECAR and OCR values are presented as the mean ± SEM from three biological replicates. * *p*<0.05, ** *p*<0.01, *** *p*<0.001.

**Supplementary Fig. S7.** **Increased PKLR and decreased ZBTB10 are associated with ADT resistance-induced PCa malignancy.** **A**, **B** Proliferation in parental LNCaP and LNCaP-MDVR cells stably transfected with the non-target control (NC) or PKLR shRNA vector (**A**) or an empty vector (EV) or ZBTB10 cDNA vector (**B**). *n* = 8 per group. * vs. parental LNCaP; ^#^ vs. the NC (**A**) or EV (**B**); by a one-way ANOVA. **C, D** Sphere formation of parental C4-2 and C4-2-MDVR cells stably expressing the NC or PKLR shRNA vector (**C**) or the EV or ZBTB10 cDNA vector (**D**). *n* = 5 per group. * vs. parental C4-2; ^#^ vs. the NC (**C**) or EV (**D**); by a two-way ANOVA. Quantification of proliferation and sphere-formation assays presented as the mean ± SEM from three biological replicates. * *p*<0.05, ** *p*<0.01, *** *p*<0.001.

**Supplementary Fig. S8. Vilanterol and saquinavir reduce cell viability in MDV3100-resistant PCa cells.** **A** Various prostatic cell lines were treated with 0, 1, 5, 10, 25, and 50 μM fosinopril (left) or salmeterol (right) for 24 h, and cell viability was determined by an MTT colorimetric assay. *n* = 8 per group. * vs. the vehicle (0 μM); by a one-way ANOVA. **B** Proliferation analysis of C4-2 cells expressing empty vector (EV) or PKLR cDNA vector, treated with 0, 1, 5, 10, 25 and 50 μM fosinopril (left) or salmeterol (right) for 24 h. *n* = 8 per group. * vs. the vehicle (0 μM); by a one-way ANOVA. **C** Proliferation analysis of C4-2 and C4-2-MDVR cells treated with 0, 1, 5, 10, 25 and 50 μM fosinopril (left) or salmeterol (right) for 24 h. *n* = 8 per group. * vs. the vehicle (0 μM); by a one-way ANOVA. **D** Proliferation analysis of LNCaP or LNCaP-MDVR cells, treated with 0, 1, 5, 10, 25 and 50 μM candidate PKLR inhibitors for 24 h. *n* = 8 per group. * vs. the vehicle (0 μM); by a one-way ANOVA. Quantification of cell viability presented as the mean ± SEM from three biological replicates. * *p*<0.05, ** *p*<0.01.

**Supplementary Fig. S9. Vilanterol and saquinavir reduce NED and glucose metabolism in PKLR-overexpressed or ADT-resistant PCa cells. A** Sphere-formation assays of C4-2 and LNCaP cells exposed to the vehicle (DMSO), vilanterol (10 μM), or saquinavir (10 μM) for 1 week. *n* = 5 per group. Quantification of sphere formation is presented as the mean ± SEM from three biological replicates. **B, C** Quantification of glucose uptake, lactate amounts, and pyruvate contents by colorimetric assays of LNCaP and C4-2 cells expressing the empty vector (EV) or PKLR cDNA vector (**B**) or following treatment of charcoal-stripped serum (CSS)-containing medium for 48 h (**C**), and treated with DMSO, 10 μM vilanterol, or 10 μM saquinavir for 24 h. * vs. DMSO; ^#^ vs. the EV (**B**) or -CSS (**C**); by a two-way ANOVA. Relative glucose uptake, lactate amounts, and pyruvate levels are presented as the mean ± SEM from three biological replicates. * *p*<0.05, ** *p*<0.01. **D** Relative ENO2 (left), NANOG (middle), and PKLR (right) mRNA levels in C4-2/PKLR, C4-2-MDVR, PC3, and LASCPC01 cells with DMSO, 10 μM vilanterol, or 10 μM saquinavir treatment for 24 h. * vs. DMSO. ** *p*<0.01, *** *p*<0.001; by a one-way ANOVA. Quantification of mRNA is presented as the mean ± SEM from three biological replicates. * *p*<0.05, ** *p*<0.01, *** *p*<0.001.

**Supplementary Fig. S10. Vilanterol and saquinavir reduce tumor growth and NED in ADT-resistant PCa cells. A, B** Tumor growth analysis of mice subcutaneously inoculated with C4-2-MDVR cells. Tumor sizes were monitored once a week (**A**), and tumor weights (**B**) were obtained at the end of the experiment (*n* = 4 mice per group). * vs. DMSO. ** *p*<0.01, *** *p*<0.001; by a one-way ANOVA. **C, D** IHC staining and representative intensity of PKLR, ENO2, MKI67, and PCNA from C4-2-MDVR subcutaneous tumors. * vs. DMSO. Significance was determined by a two-tailed Student’s *t*-test. ** *p*<0.01, *** *p*<0.001.

**Supplementary Fig. S11. Loss of ZBTB10 in SCPC samples.** IHC staining (left) and relative intensity of PKLR and ZBTB10 (right, by H-score analysis) in SCPC samples (*n*=8) from the Duke University School of Medicine. Scale bars, 100 μm. Data are presented as the mean ± SEM. * vs. PKLR. **** *p*<0.0001; by a two-tailed Student’s *t*-test.

**Supplementary Tables**

**Supplementary Table S1.** **Primer sequences of the constructs.** Primer sequences of the wild-type ZBTB10-binding elements of human *PKLR* regulatory sequence reporter (WT-*PKLR*) and the ZBTB10-binding element mutants of the human *PKLR* regulatory sequence reporter (ZREM1~4) are listed 5’-3’. WT, wild-type; M, mutant; F, forward; R, reverse).

| Promoter reporter | Sequence |
| --- | --- |
| *PKLR*/ZRE-WT F | GAGCAACTGAACCAAAGCCT |
| *PKLR*/ZRE-WT R | TCCTCCTATGTTCCATGGCT |
| *PKLR*/ZRE1M F | TCCTCAGTCAGCACGAATTCGATCTGGGAGA |
| *PKLR*/ZRE1M R | TGCTGACTGAGGATGAGATGGG |
| *PKLR*/ZRE2M F | AAGTACCCCAGGATGAATTCCTGAAAGGACC |
| *PKLR*/ZRE2M R | TCCTGGGTACTTTACTCTGT |
| *PKLR*/ZRE3M F | ACTACTGTGTCCGAATTCCCTGATACAG |
| *PKLR*/ZRE3M R | GGACACAGTAGTGAGCAAAA |
| *PKLR*/ZRE4M F | TCCATCCTGAGAATTCCTCCAGGAGGT |
| *PKLR*/ZRE4M R | TCAGGATGGACTTTGCTAA |

**Supplementary Table S2. RT-qPCR primer sequences.** Sequences of the primers used in real-time qPCR assays in this study are listed 5’-3’. F, forward; R, reverse; m, murine.

| Gene | Primer sequence | Gene | Primer sequence |
| --- | --- | --- | --- |
| *PKLR* F | CGGAAGGACACGGCATCAAG | *MYCN* F | ACAGTCATCTGTCTGGACGC |
| *PKLR* R | AGCCAGGAAAACCTTCTCTGC | *MYCN* R | TGTCCTCGGATGGCTACAGT |
| *CHGA* F | ACTGAAGGAGCTCCAAGAC | *NANOG* F | AAGGTCCCGGTCAAGAAACA |
| *CHGA* R | TCTGCCTCCTTGGAATCCTC | *NANOG* R | CTTCTGCGTCACACCATTGC |
| *CHGB* F | GCCACGTGCCTATTTCATGT | *SOX2* F | ACCAGCTCGCAGACCTACAT |
| *CHGB* R | GCTCCTTCCTCACCGTAGTT | *SOX2* R | CCTGCTGCGAGTAGGACAT |
| *ENO2* F | TCAGGGACTACCTGTGGTCT | *KLK3* F | TTTCCAATGACGTCTGTGCG |
| *ENO2* R | TTCCACTGCCGCTCAATAC | *KLK3* R | CCAGAATCACCCGAGCAGG |
| *SYP* F | GGCTTTGTGAAGGTGCTGC | *NKX3-1* F | CAGAGACCGAGCCAGAAACG |
| *SYP* R | CACTCTCGGTCTTGTTGGCA | *NKX3-1* R | CTGAGTGTGGGAGAAGGCAG |
| *PCNA F* | TAATGTCGATAAAGAGGAGG | *SNAI2 F* | TTTTCCAGACCCTGGTTGCTT |
| *PCNA R* | GTGTCACCGTTGAAGAGAGTG | *SNAI2 R* | GAGCCCTCAGATTTGACCTGT |
| *MKI67 F* | GAAAGAGTGGCAACCTGCCTT | *TWIST1 F* | GCCAGGTACATCGACTTCCTC |
| *MKI67 R* | CACCAAGTTTTACTACATCTG | *TWIST1 R* | TCCATCCTCCAGACCGAGAAG |
| *SNAI1 F* | GTTTACCTTCCAGCAGCCCT | *VIM F* | GCAAAGCAGGAGTCCACTGA |
| *SNAI1 R* | TCCCAGATGAGCATTGGCAG | *VIM R* | CATTTCACGCATCTGGCGTTC |
| *ZBTB10* F | TGGTTTGATGCCTGGTCCTTC | *GAPDH* F | CCAGTAGAGGCAGGGATGAT |
| *ZBTB10* R | CCTGTGTGAATGAGCAAGTGC | *GAPDH* R | CTTTCATTGTCTTTTCCGCC |

**Supplementary Table S3.** **Western blotting antibodies.** Source and dilution of each antibody used for Western blotting in this study are listed.

| Primary antibody | Clonality | Source  (cat. no.) | Dilution | Secondary antibody | Source | Dilution |
| --- | --- | --- | --- | --- | --- | --- |
| PKLR | Polyclonal | Abcam (ab125697) | 1:1000 | anti-rabbit IgG | Jackson Labs | 1:20000 |
| ENO2 | Monoclonal | Santa Cruz  (sc-21738) | 1:100 | anti-mouse IgG | Jackson Labs | 1:5000 |
| CHGA | Monoclonal | Santa Cruz  (sc-393941 ) | 1:100 | anti-mouse IgG | Jackson Labs | 1:5000 |
| ZBTB10 | Polyclonal | Abcam (ab117786) | 1:2000 | anti-rabbit IgG | Jackson Labs | 1:20000 |
| KLK3 | Monoclonal | Santa Cruz  (sc-7316) | 1:200 | anti-mouse IgG | Jackson Labs | 1:5000 |
| NKX3-1 | Monoclonal | Thermo-Fisher (MA5-15618) | 1:2000 | anti-mouse IgG | Jackson Labs | 1:5000 |
| β-actin | Polyclonal | GeneTex (GTX109639) | 1:1000 | anti-rabbit IgG | Jackson Labs | 1:20000 |

**Supplementary Table S4.** **ChIP antibodies and primer sequences.** Source and dilution of each antibody and the sequences (5’-3’) of each primer used for ChIP in this study are listed.

| ChIP antibodies | | | | | |
| --- | --- | --- | --- | --- | --- |
| Primary antibody | Species | | Clonality | Source | Dilution |
| ZBTB10 | Rabbit | | Polyclonal | Abcam (ab117786) | 1:50 |
| Acety-H3 | Rabbit | | Monoclonal | Novus (NB300-221) | 1:100 |
| IgG | Rabbit | |  | Santa Cruz (sc-2027) | 1:50 |
| ChIP primers | | | | | |
| Site | | Sequence | | | |
| *PKLR*/ZRE1 F | | GAGCAACTGAACCAAAGCCT | | | |
| *PKLR*/ZRE1 R | | CCTTTGCTCCTCCCTCAGAA | | | |
| *PKLR*/ZRE2 F | | TATGGCTGGGTGACTGAGAC | | | |
| *PKLR*/ZRE2 R | | TCCTACCTCCTAGAGCCTCA | | | |
| *PKLR*/ZRE3 F | | AAAGGCCCTGTCTGTTTTGC | | | |
| *PKLR*/ZRE3 R | | AACTGGTGGGATTCTCTGGA | | | |
| *PKLR*/ZRE4 F | | CCACACTGAAAGCATGTCG | | | |
| *PKLR*/ZRE4 R | | TCCTCCTATGTTCCATGGCT | | | |

**Reference**

1. Gutierrez-Guerrero A, Cosset FL, Verhoeyen E. Lentiviral Vector Pseudotypes: Precious Tools to Improve Gene Modification of Hematopoietic Cells for Research and Gene Therapy. *Viruses* 2020, **12**(9): e1016.

2. Taylor BS, Schultz N, Hieronymus H, Gopalan A, Xiao Y, Carver BS*, et al.* Integrative genomic profiling of human prostate cancer. *Cancer Cell* 2010, **18**(1)**:** 11-22.

3. Cancer Genome Atlas Research N. The Molecular Taxonomy of Primary Prostate Cancer. *Cell* 2015, **163**(4)**:** 1011-1025.

4. Beltran H, Prandi D, Mosquera JM, Benelli M, Puca L, Cyrta J*, et al.* Divergent clonal evolution of castration-resistant neuroendocrine prostate cancer. *Nat Med* 2016, **22**(3)**:** 298-305.

5. Aggarwal R, Huang J, Alumkal JJ, Zhang L, Feng FY, Thomas GV*, et al.* Clinical and Genomic Characterization of Treatment-Emergent Small-Cell Neuroendocrine Prostate Cancer: A Multi-institutional Prospective Study. *J Clin Oncol* 2018, **36**(24)**:** 2492-2503.

6. Wang G, Jones SJ, Marra MA, Sadar MD. Identification of genes targeted by the androgen and PKA signaling pathways in prostate cancer cells. *Oncogene* 2006, **25**(55)**:** 7311-7323.

7. Nelson PS, Clegg N, Arnold H, Ferguson C, Bonham M, White J*, et al.* The program of androgen-responsive genes in neoplastic prostate epithelium. *Proc Natl Acad Sci U S A* 2002, **99**(18)**:** 11890-11895.

8. Subramanian A, Tamayo P, Mootha VK, Mukherjee S, Ebert BL, Gillette MA*, et al.* Gene set enrichment analysis: a knowledge-based approach for interpreting genome-wide expression profiles. *Proc Natl Acad Sci U S A* 2005, **102**(43)**:** 15545-15550.

9. Yang JM, Chen CC. GEMDOCK: a generic evolutionary method for molecular docking. *Proteins* 2004, **55**(2)**:** 288-304.

10. Valentini G, Chiarelli LR, Fortin R, Dolzan M, Galizzi A, Abraham DJ*, et al.* Structure and function of human erythrocyte pyruvate kinase. Molecular basis of nonspherocytic hemolytic anemia. *J Biol Chem* 2002, **277**(26)**:** 23807-23814.

11. The UniProt C. UniProt: the universal protein knowledgebase. *Nucleic Acids Res* 2017, **45**(D1)**:** D158-D169.

12. Goodsell DS, Zardecki C, Di Costanzo L, Duarte JM, Hudson BP, Persikova I*, et al.* RCSB Protein Data Bank: Enabling biomedical research and drug discovery. *Protein Sci* 2020, **29**(1)**:** 52-65.

13. Guex N, Peitsch MC. SWISS-MODEL and the Swiss-PdbViewer: an environment for comparative protein modeling. *Electrophoresis* 1997, **18**(15)**:** 2714-2723.
