# Supplementary figures and images for: Pyruvate kinase L/R links metabolism dysfunction to neuroendocrine differentiation of prostate cancer by ZBTB10 deficiency
Source: Cell Death Dis. 2022 Mar 19;13(3):252. doi: 10.1038/s41419-022-04694-z (PMC8934352; doi:10.1038/s41419-022-04694-z)

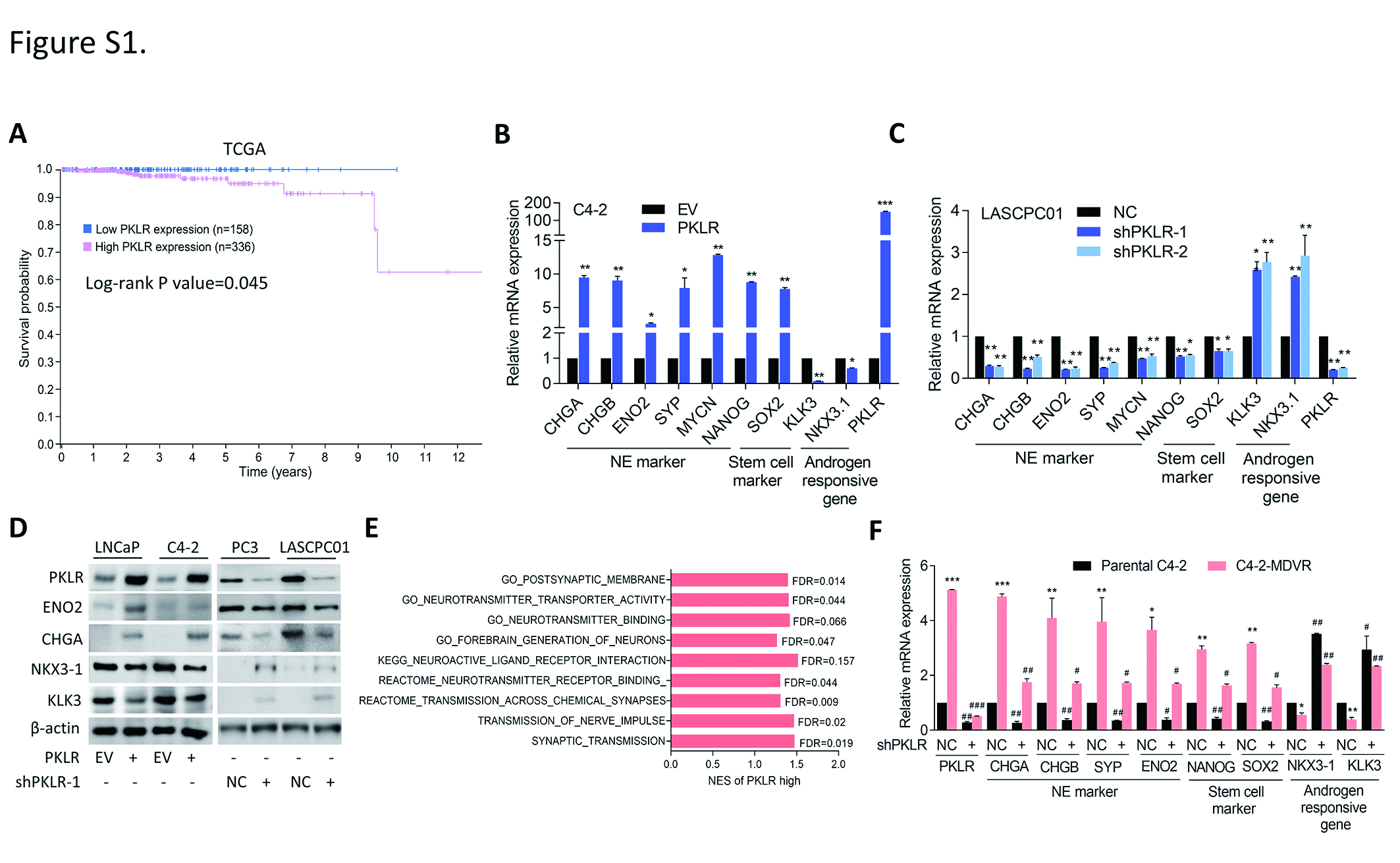

Supplement: Supplementary file 2 — Supplementary Fig. S1 [file 41419_2022_4694_MOESM2_ESM.tif]

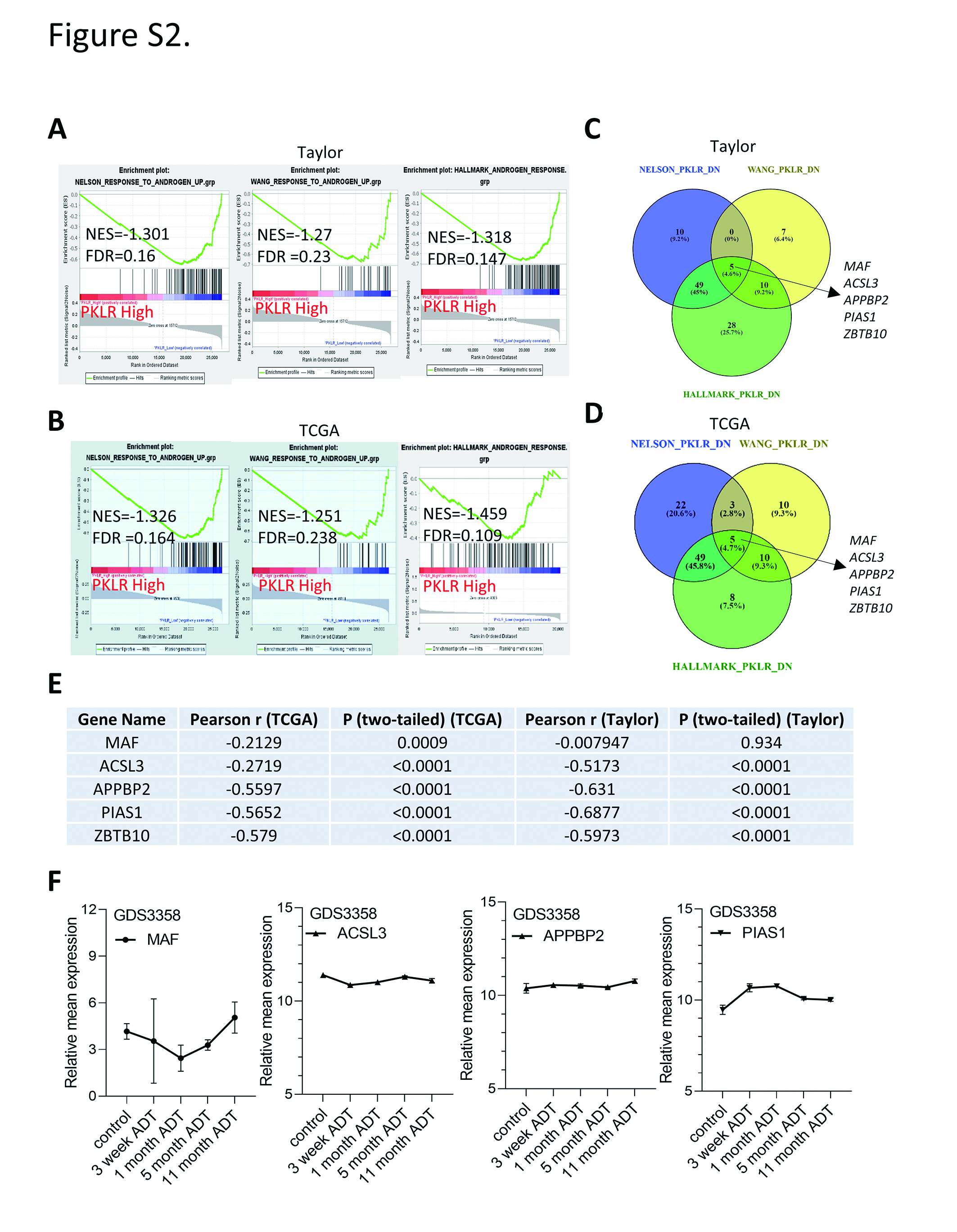

Supplement: Supplementary file 3 — Supplementary Fig. S2 [file 41419_2022_4694_MOESM3_ESM.tif]

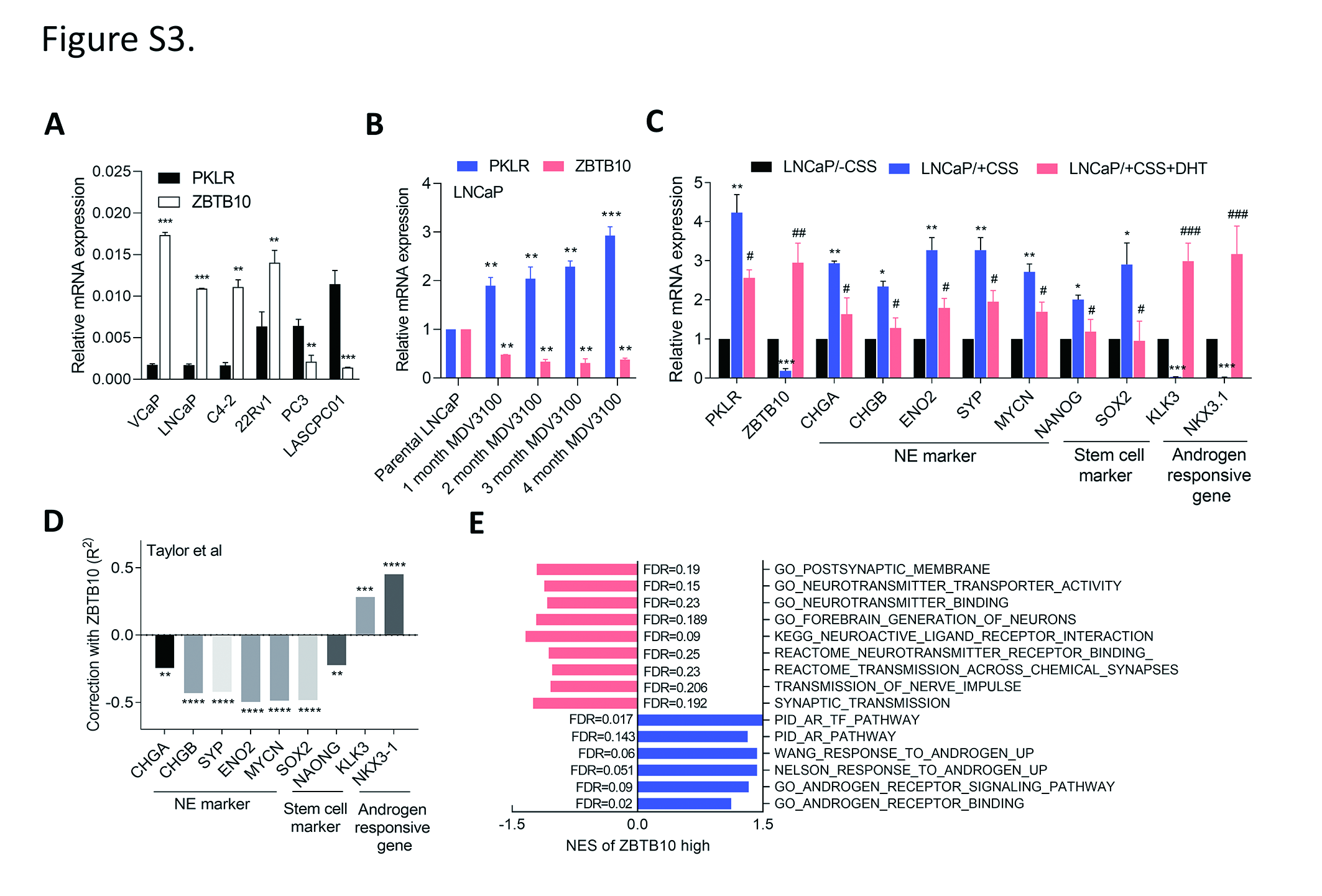

Supplement: Supplementary file 4 — Supplementary Fig. S3 [file 41419_2022_4694_MOESM4_ESM.tif]

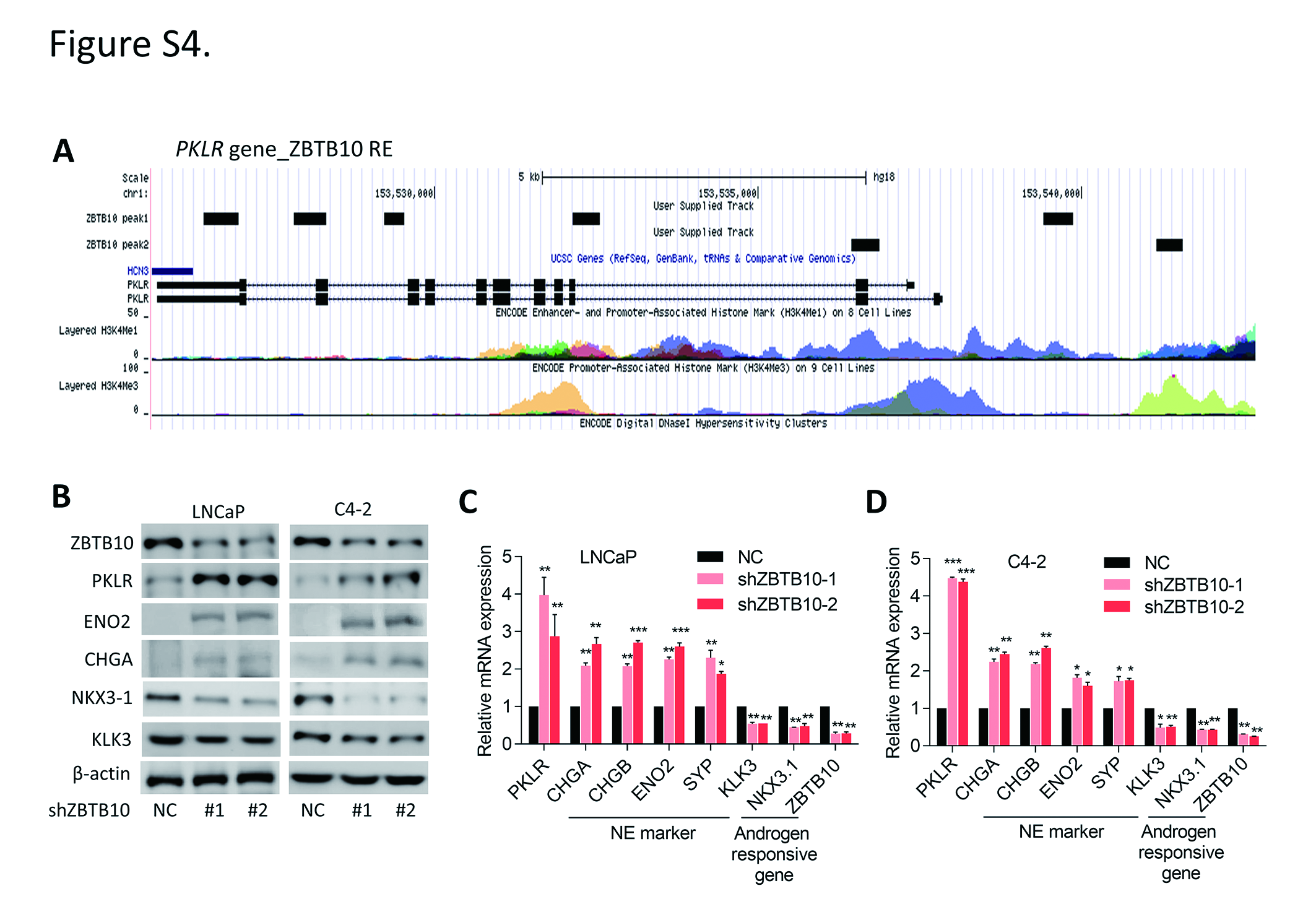

Supplement: Supplementary file 5 — Supplementary Fig. S4 [file 41419_2022_4694_MOESM5_ESM.tif]

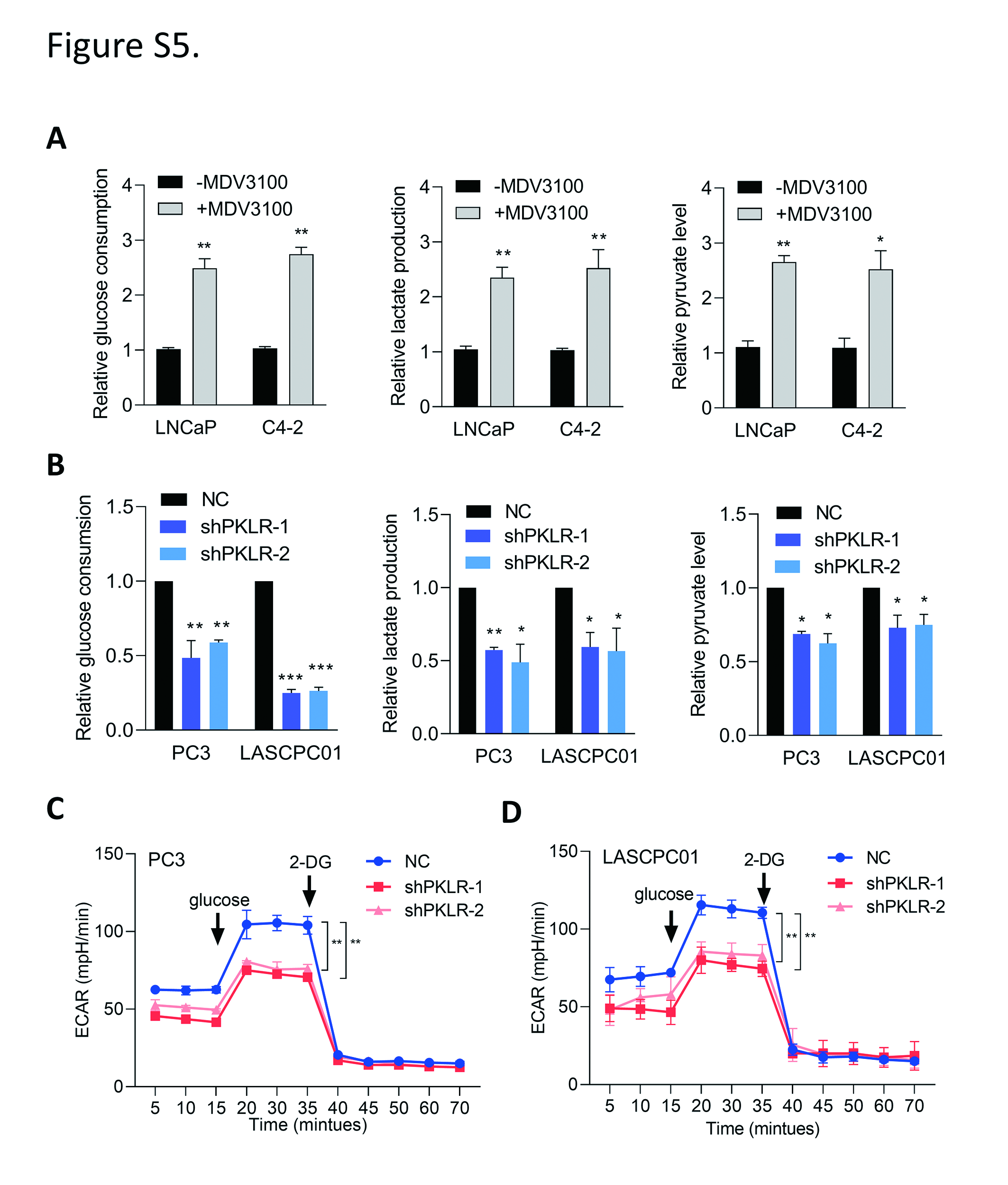

Supplement: Supplementary file 6 — Supplementary Fig. S5 [file 41419_2022_4694_MOESM6_ESM.tif]

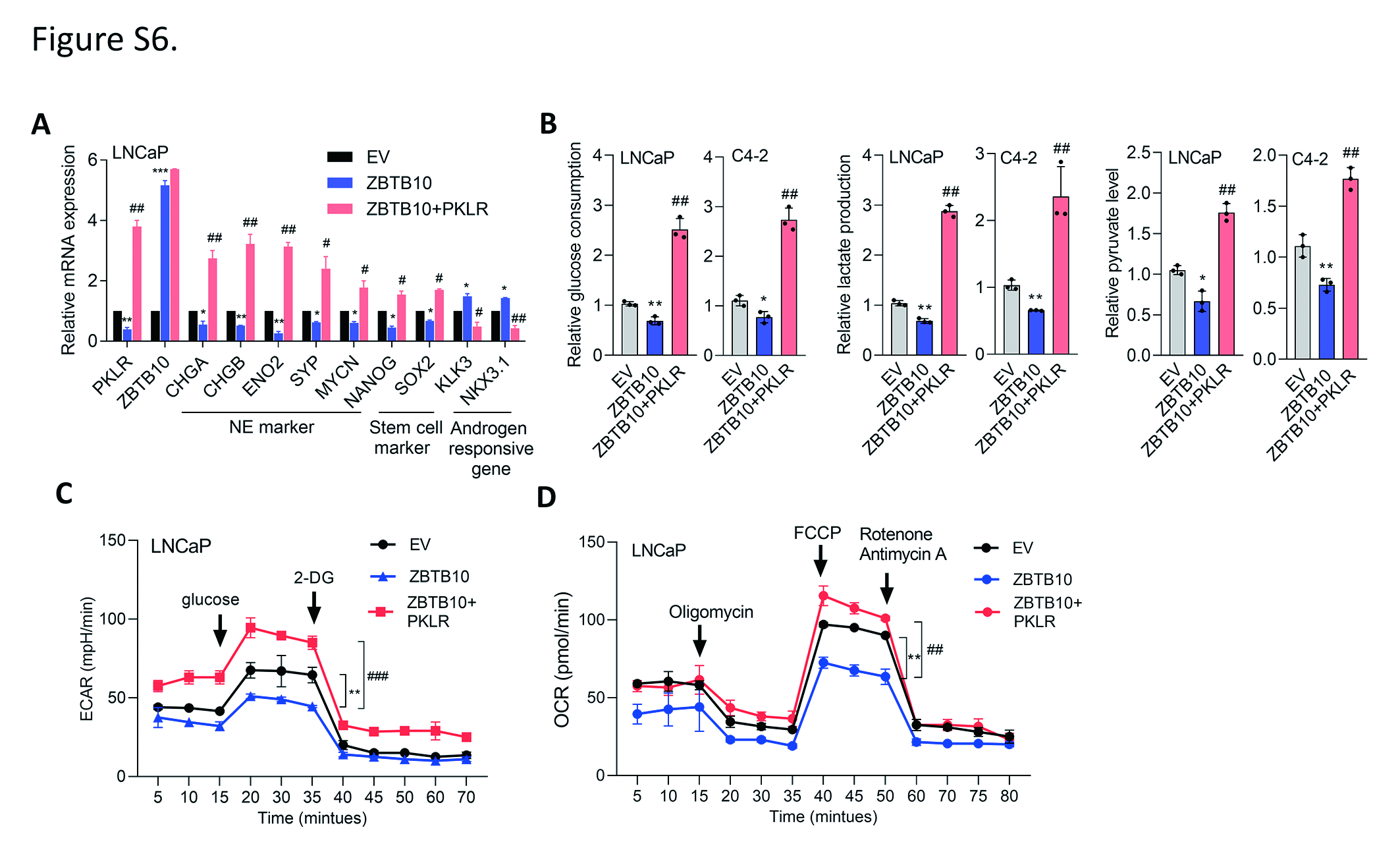

Supplement: Supplementary file 7 — Supplementary Fig. S6 [file 41419_2022_4694_MOESM7_ESM.tif]

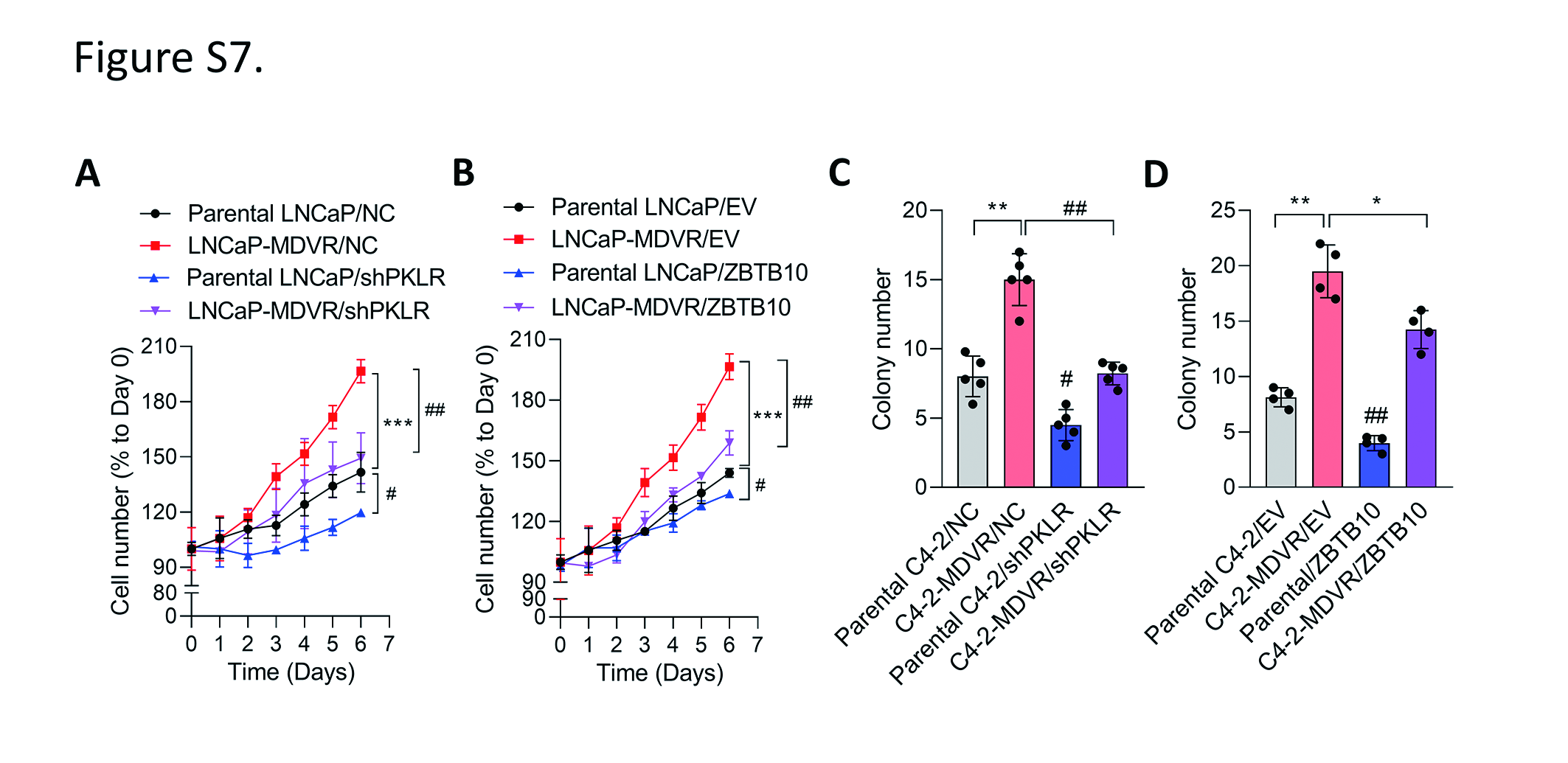

Supplement: Supplementary file 8 — Supplementary Fig. S7 [file 41419_2022_4694_MOESM8_ESM.tif]

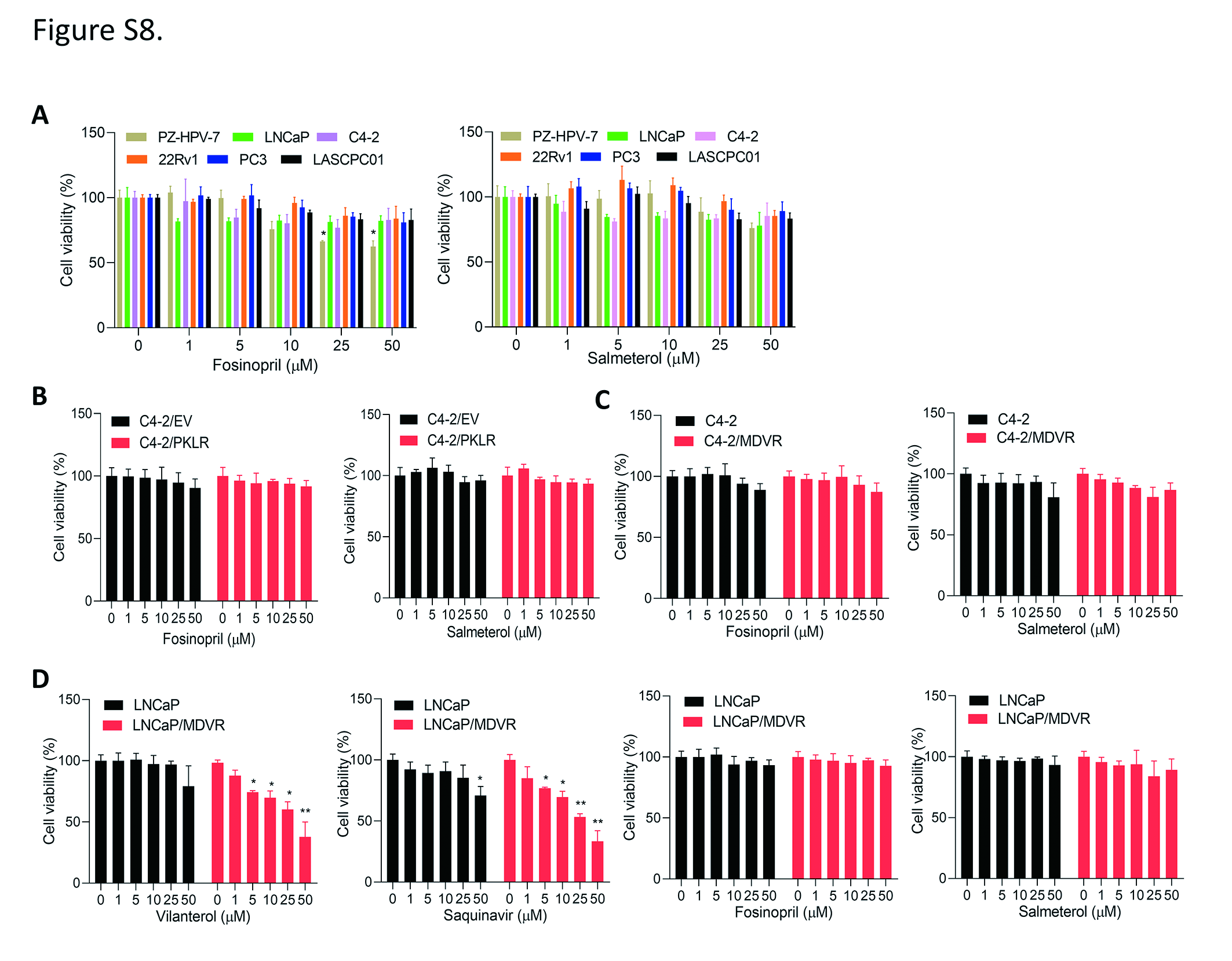

Supplement: Supplementary file 9 — Supplementary Fig. S8 [file 41419_2022_4694_MOESM9_ESM.tif]

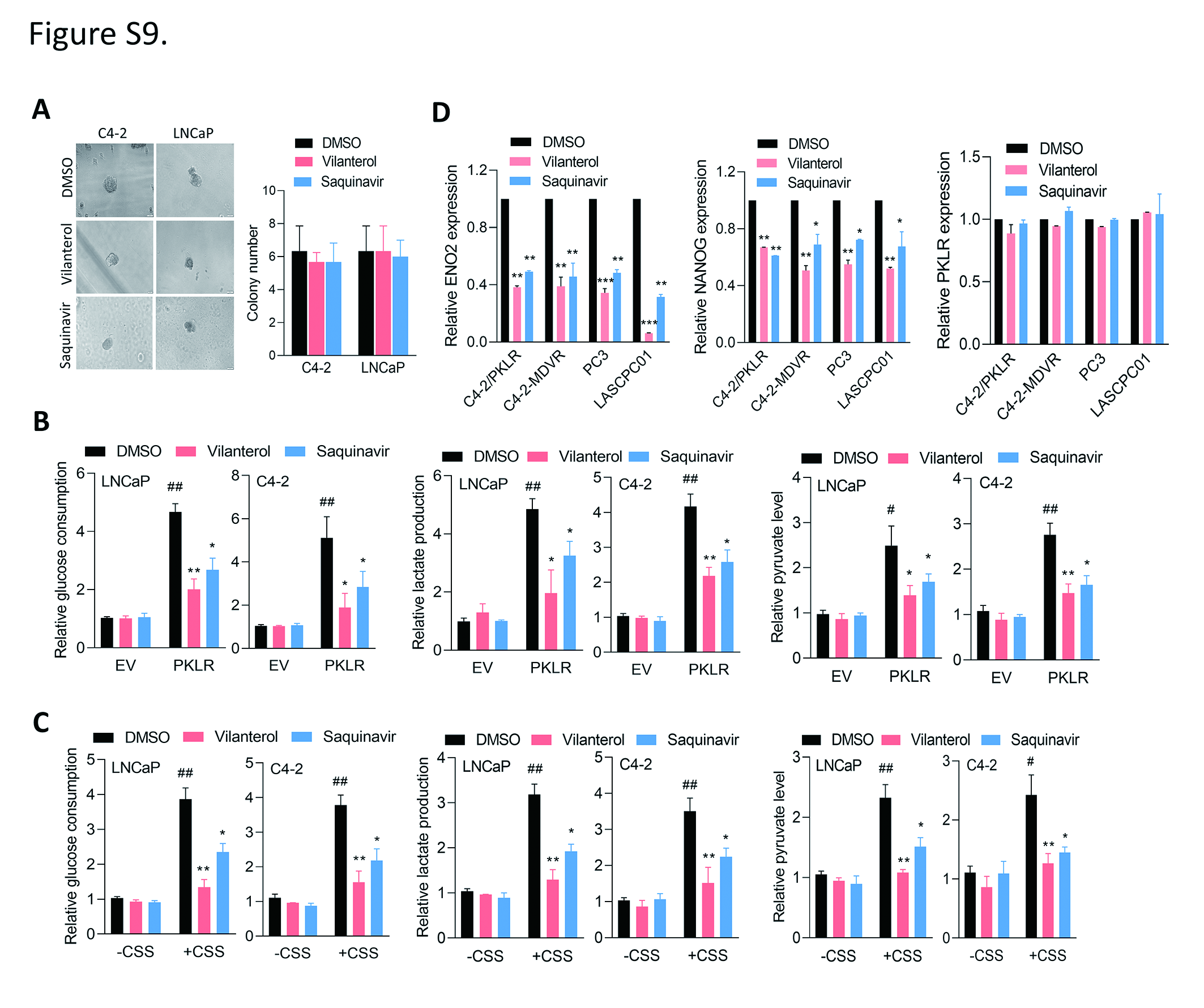

Supplement: Supplementary file 10 — Supplementary Fig. S9 [file 41419_2022_4694_MOESM10_ESM.tif]

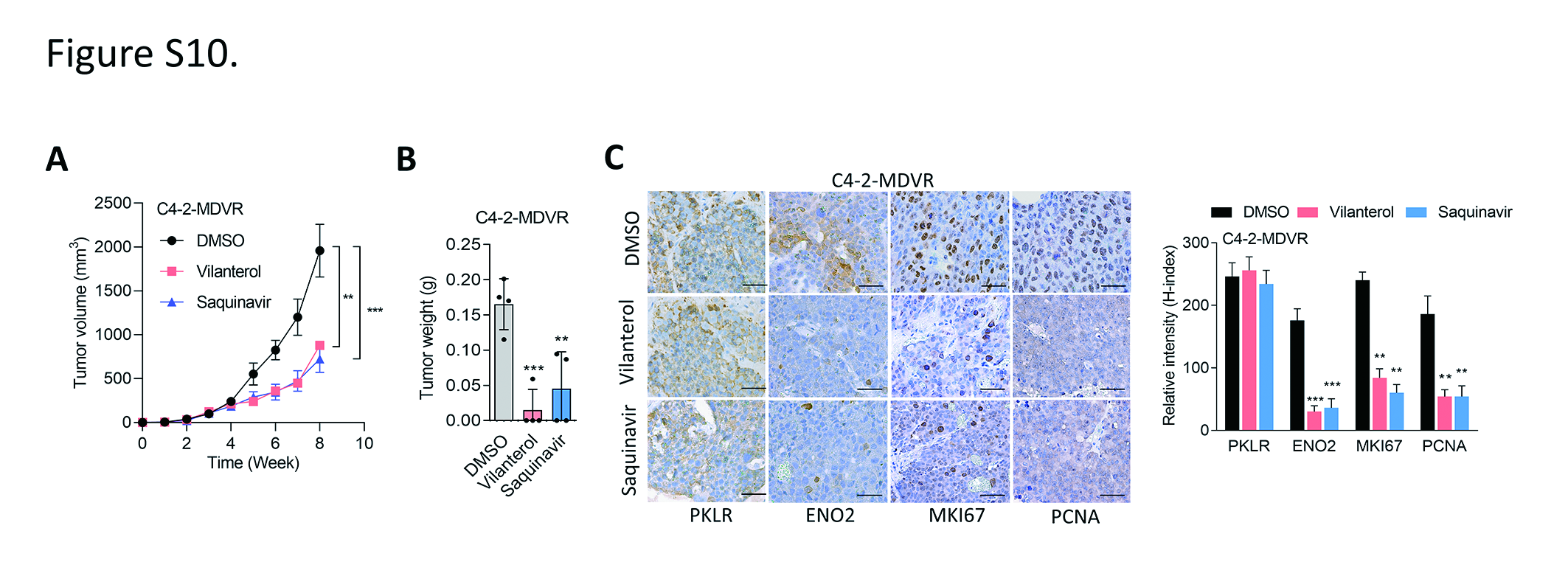

Supplement: Supplementary file 11 — Supplementary Fig. S10 [file 41419_2022_4694_MOESM11_ESM.tif]

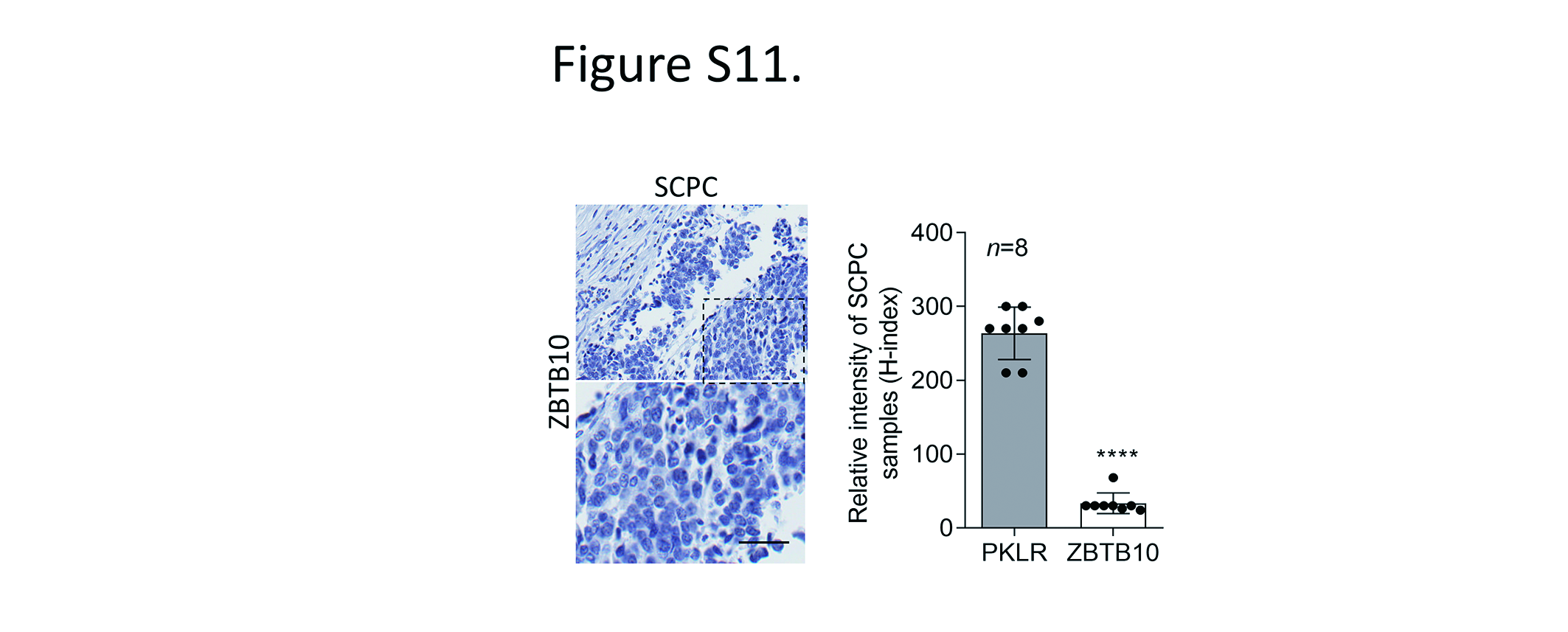

Supplement: Supplementary file 12 — Supplementary Fig. S11 [file 41419_2022_4694_MOESM12_ESM.tif]

Figure 1H

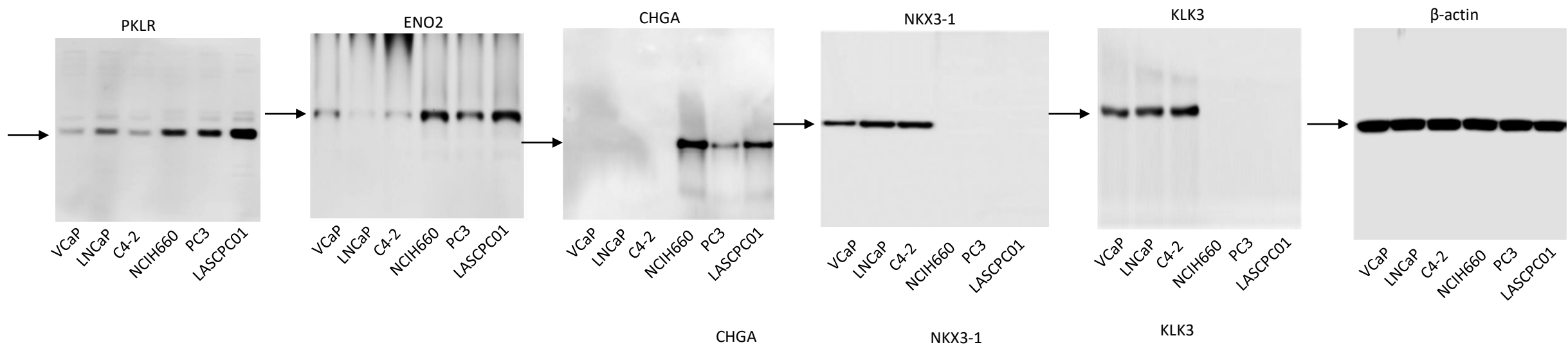

Figure 1K

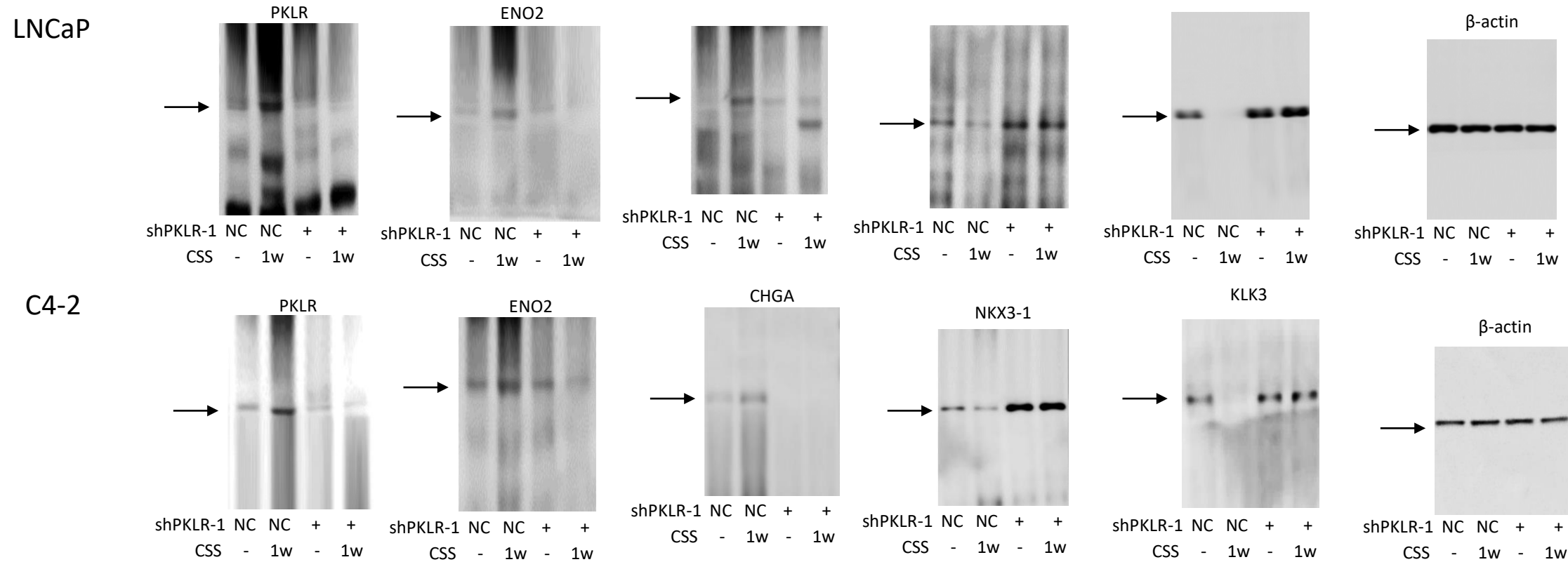

Figure 2C

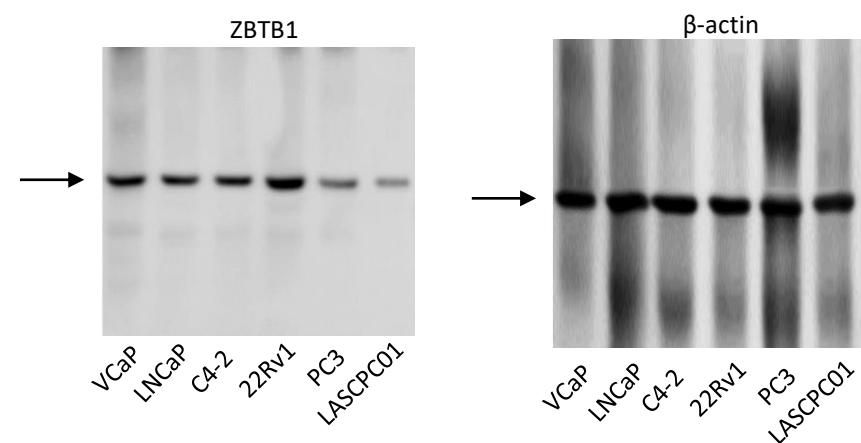

Figure 2F

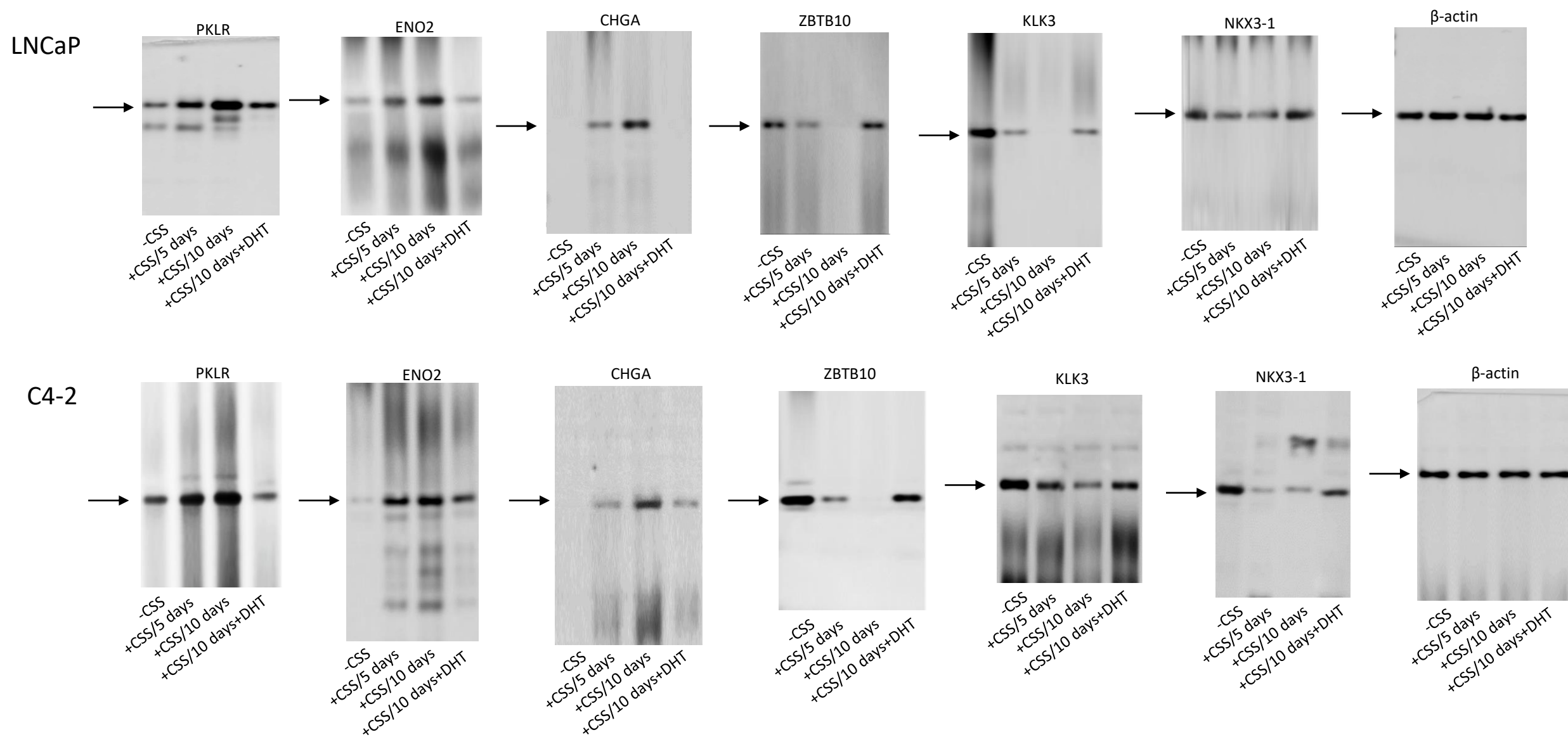

Figure 3A

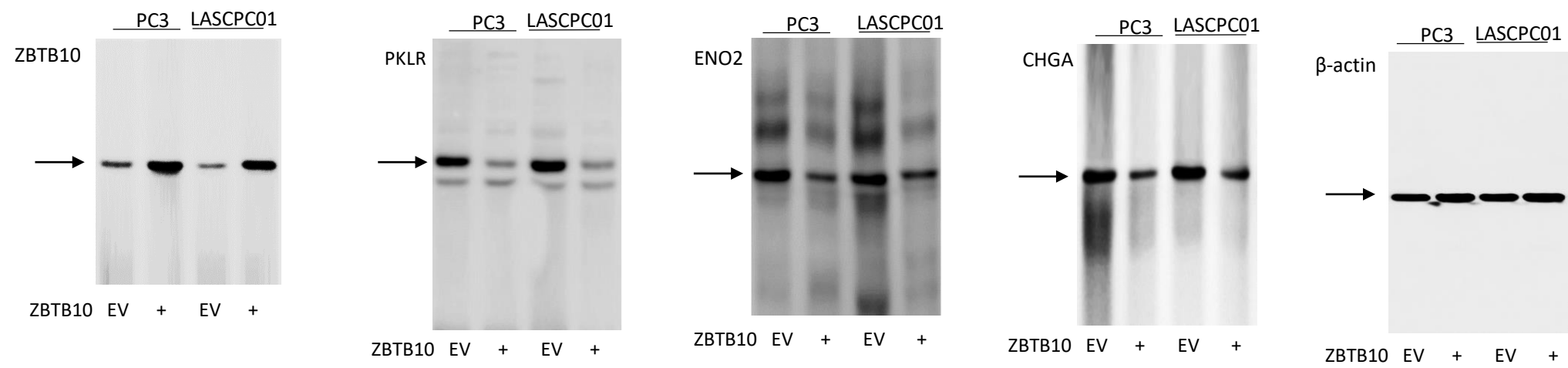

Figure 4K

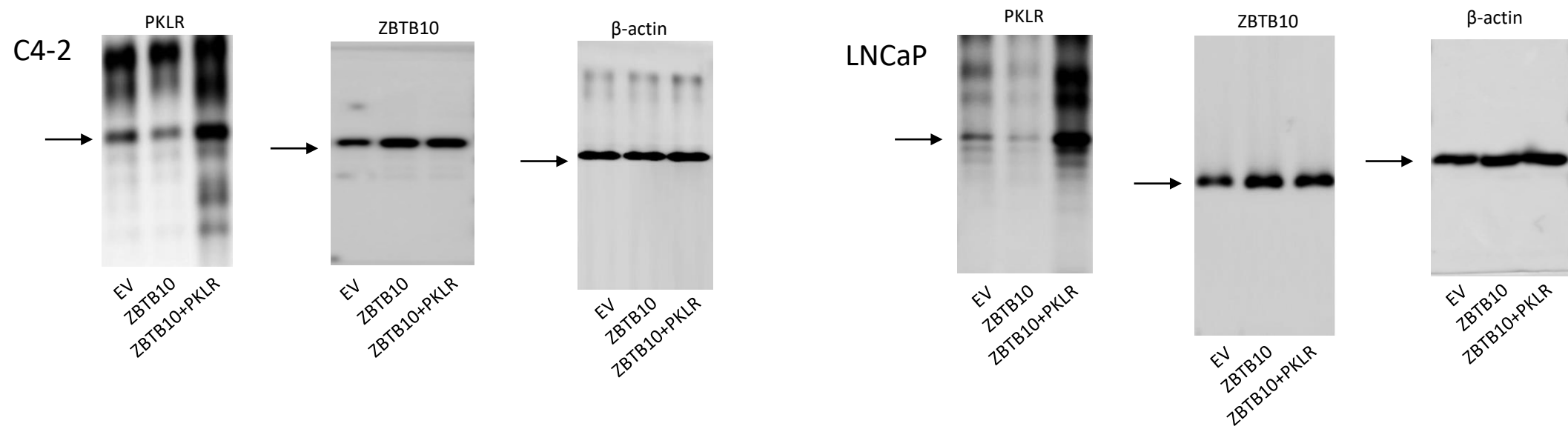

Figure 5E

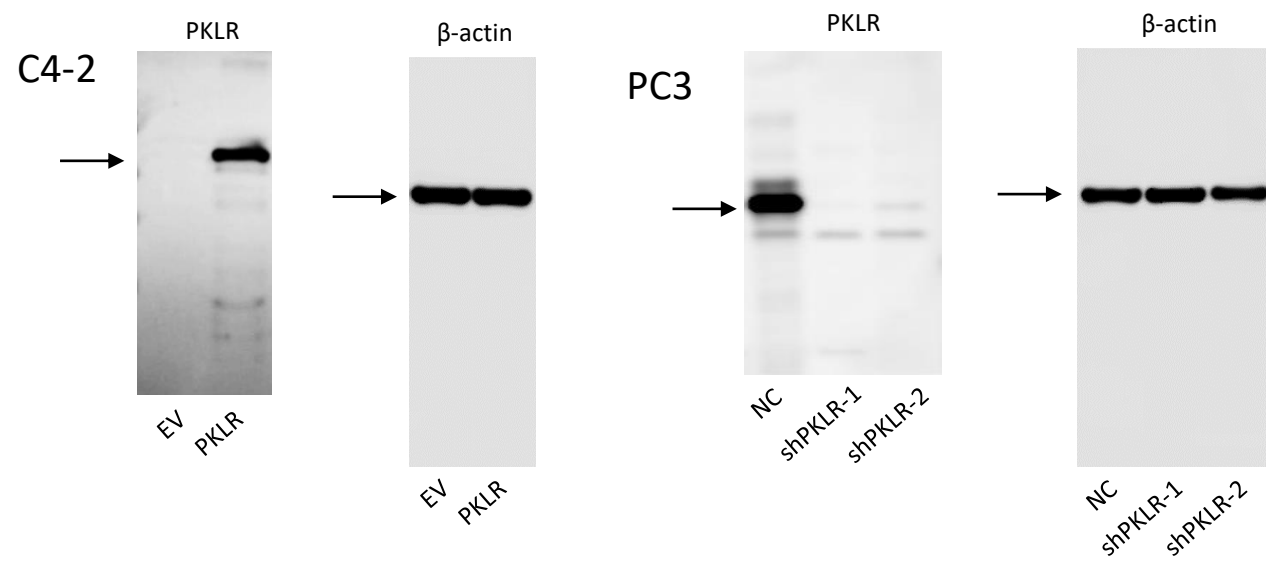

Supplement: Supplementary file 13 — Original Data File [file 41419_2022_4694_MOESM13_ESM.pdf]
